# Type I Interferons Promote Fatal Immunopathology by Regulating Inflammatory Monocytes and Neutrophils during *Candida* Infections

**DOI:** 10.1371/journal.ppat.1002811

**Published:** 2012-07-26

**Authors:** Olivia Majer, Christelle Bourgeois, Florian Zwolanek, Caroline Lassnig, Dontscho Kerjaschki, Matthias Mack, Mathias Müller, Karl Kuchler

**Affiliations:** 1 Medical University Vienna - Max F. Perutz Laboratories, Christian Doppler Laboratory for Infection Biology, Campus Vienna Biocenter, Vienna, Austria; 2 University of Veterinary Medicine Vienna, Institute of Animal Breeding and Genetics & Biomodels Austria, Vienna, Austria; 3 Medical University of Vienna – Clinical Institute of Pathology, AKH - General Hospital of Vienna, Vienna, Austria; 4 University Hospital of Regensburg, Internal Medicine II – Nephrology, Regensburg, Germany; University of Wisconsin-Madison, United States of America

## Abstract

Invasive fungal infections by *Candida albicans* (Ca) are a frequent cause of lethal sepsis in intensive care unit patients. While a contribution of type I interferons (IFNs-I) in fungal sepsis remains unknown, these immunostimulatory cytokines mediate the lethal effects of endotoxemia and bacterial sepsis. Using a mouse model lacking a functional IFN-I receptor (*Ifnar1^−/−^*), we demonstrate a remarkable protection against invasive Ca infections. We discover a mechanism whereby IFN-I signaling controls the recruitment of inflammatory myeloid cells, including Ly6C^hi^ monocytes and neutrophils, to infected kidneys by driving expression of the chemokines CCL2 and KC. Within kidneys, monocytes differentiate into inflammatory DCs but fail to functionally mature in *Ifnar1^−/−^* mice, as demonstrated by the impaired upregulation of the key activation markers PDCA1 and iNOS. The increased activity of inflammatory monocytes and neutrophils results in hyper-inflammation and lethal kidney pathology. Pharmacological diminution of monocytes and neutrophils by treating mice with pioglitazone, a synthetic agonist of the nuclear receptor peroxisome proliferator-activated receptor-γ (PPAR-γ), strongly reduces renal immunopathology during Ca infection and improves mouse survival. Taken together, our data connect for the first time the sepsis-promoting functions of IFNs-I to the CCL2-mediated recruitment and the activation of inflammatory monocytes/DCs with high host-destructing potency. Moreover, our data demonstrate a therapeutic relevance of PPAR-γ agonists for microbial infectious diseases where inflammatory myeloid cells may contribute to fatal tissue damage.

## Author Summary

Inflammation constitutes a major host response in many microbial infections. Innate immune cells orchestrate the inflammatory response to kill pathogens and clear infections. However, invasive infections by pathogenic microbes including the fungus *Candida albicans*, can result in an uncontrolled hyper-inflammatory response, leading to severe host damage and sepsis. Type I interferons constitute a hallmark of protective innate immunity in viral and bacterial infections, but at the same time have been notoriously known for their sepsis-promoting effects in numerous experimental inflammation models. Here, we show that type I interferon-signaling mediates the lethal hyper-inflammatory response during systemic mouse infections with *C. albicans*. Following fungal infections, type I interferons promote the recruitment and activation of inflammatory monocytes and neutrophils to infected organs. The high abundance and activity of inflammatory phagocytes lead to fatal tissue damage. Remarkably, we show that the pharmacological suppression of these inflammatory cells with the drug pioglitazone reduces immunopathology and sepsis-related lethality, suggesting a novel therapeutic option to combat fungal sepsis. In conclusion, our data couple the sepsis-promoting role of type I interferons to the host-destructive activity of inflammatory monocytes and neutrophils. We propose that therapeutic approaches dampening hyper-inflammation might be of general importance in microbial diseases where deleterious immunopathology occurs.

## Introduction

Fungal sepsis is a frequent cause of death in the intensive care unit, with *Candida* spp. being among the most common microbial pathogens isolated from septic patients. Worldwide, *C. albicans* (Ca) represents the most prevalent isolate recovered from human candidemic patients. The inflammatory response and disease progression of invasive candidiasis presents a similar clinical picture as seen for bacterial sepsis, with some patients developing septic shock and organ dysfunction, the most common of which is acute renal failure [1].

Type I interferons (IFNs-I) constitute a family of pleiotropic cytokines that regulate resistance to viruses, enhance innate and adaptive immunity, and modulate cell survival and apoptosis [2]. The most relevant members include IFN-β, with only one member in humans and mice, and the IFN-α family encompassing more than 10 members. While IFNs-I strongly protect against viral infections, they can have either protective or detrimental host effects in bacterial infections depending on the pathogen in question [3]. In comparison, little is known about their contributions in fungal infections. We and others have recently reported that mouse bone marrow-derived dendritic cells (BM-DCs) produce IFN-β in response to *Candida* spp. *in vitro* [4], [5]. Notably, the IFN-I receptor subunit IFNAR1 is among the highest upregulated genes in blood leukocytes in a mouse model of invasive candidiasis [6]. Furthermore, similar to bacterial infections, recent reports suggest that IFNs-I are implicated in the *in vivo* response to fungal pathogens, albeit with opposing effects for the host [7], [8]. The divergent functions of IFNs-I may relate to their versatile effects on antimicrobial immunity and to their ability to trigger either inflammatory or anti-inflammatory responses depending on the particular pathological situation [9].

Besides modulating innate and adaptive immune responses, IFNs-I play a critical role in promoting lethal endotoxemia and sepsis. For instance, mice lacking the IFN-I receptor (*Ifnar1^−/−^*) are highly insensitive to LPS- or TNF-induced lethal shock [10], [11]. Notably, they also show improved survival in a model of septic peritonitis [12]. However, the concept of IFNs-I as adverse mediators of sepsis has been challenged by a recent study using a low lethality model of cecal ligation and puncture-induced sepsis [13]. In this model, *Ifnar1^−/−^* mice show an increased late mortality, underlining the complex effects of IFNs-I. The apparent discrepancies might relate to differences in the temporal regulation of the IFN-I response, the amount or subtype of cytokines produced or the microbial species used within a certain model [14].

This work addresses the pathophysiological role of IFN-I signaling during Ca infections using an intravenous (iv) mouse challenge model. As in humans, the mouse kidney is the prime target organ, as progressive sepsis concomitant with renal failure account for mortality in that model [15]. Since the severity of kidney tissue damage is quantitatively related to the level of host innate response, it has been suggested that an uncontrolled inflammatory response rather than Ca itself may worsen disease outcome. Indeed, massive infiltrations of neutrophils are commonly observed and believed to contribute significantly to host tissue destruction [16].

Another highly inflammatory cell type frequently associated with host immunopathologies are Ly6C^hi^ inflammatory monocytes [17]–[19]. The chemokine CCL2 recruits inflammatory monocytes to infected body sites, where they exert direct anti-microbial activities or further differentiate into inflammatory DCs [20]. This DC subtype participates in the protective innate response and is characterized by high production of TNF-α and iNOS [21]. Interestingly, IFNs-I have been shown to regulate Ly6C^hi^ monocyte recruitment during viral infections [22], [23], as well as during chronic inflammation in mice [19] by inducing *Ccl2*. However, the role of inflammatory monocytes in the host response to Ca infections remains unknown.

Here, we demonstrate a pivotal role of IFNs-I in triggering the inflammatory host response and immunopathology in experimental candidiasis. We establish a mechanism through which IFNs-I mediate the lethal effects of Ca-induced sepsis. IFN-I signaling stimulates the recruitment of both Ly6C^hi^ inflammatory monocytes and neutrophils from the bone marrow to infected kidneys. Furthermore, IFN-I signaling is required for the subsequent maturation of monocytes into functional inflammatory DCs. Thus, mice competent for IFN-I signaling (WT) suffer from unrestrained hyper-inflammation, resulting in lethal kidney pathology. In sharp contrast, *Ifnar1^−/−^* mice are remarkably resistant to otherwise lethal Ca infections, and show reduced activity of inflammatory myeloid cells. Strikingly, the pharmacological suppression of inflammatory monocytes and neutrophils by the anti-diabetes drug pioglitazone, a PPAR-γ agonist, strongly reduces renal immunopathology and improves survival of mice, suggesting a novel therapeutic option to combat fungal sepsis. Our data provide a molecular mechanism explaining the manifestation and progression of systemic fungal infections. We suggest that interfering with the activity of inflammatory monocytes and neutrophils provides beneficial effects for disease outcome by suppressing fatal kidney pathology during Ca infections. Importantly, our results are the first report of a central role of the IFN-I-driven CCL2/CCR2 pathway in controlling inflammatory monocyte trafficking during fungal infections.

## Results

### Detrimental role of IFN-I signaling in a *Candida* intravenous infection mouse model

**Figure 1:**
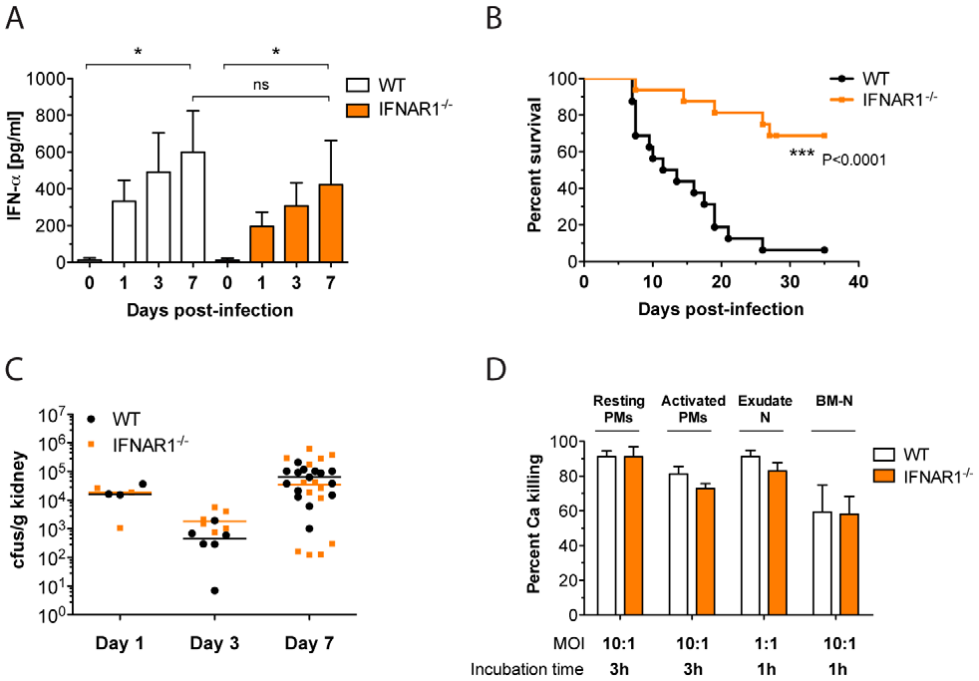
Ca induces a detrimental IFN-I response during infection. (A) Mice of the indicated genotype were iv injected with a lethal dose of 1×10^5^ cfus Ca. Serum was collected at indicated time points, and IFN-α concentrations were determined using a multiplex bead array system. Data presented show the mean ± SEM of two independent experiments (n = 6–8 mice per group). (B) Mice were injected as in (A) and survival was monitored for 35 days. The data are presented as Kaplan-Meier survival curves and are a summary of three independent experiments (n = 16 mice per group). (C) Mice were injected with 0.5×10^5^ cfus Ca. At indicated time points, Ca cfus in kidneys were determined and expressed as cfus/g organ. Data presented are a summary of three independent experiments (n = 3–15 mice per group). Each symbol represents one mouse; horizontal bars indicate the calculated median. (D) Resting and activated peritoneal macrophages (PMs), exudate neutrophils (N), and bone marrow neutrophils (BM-N) were stimulated with Ca at the indicated multiplicities of infection and for indicated time periods. Percentage of Ca killing was determined by counting cfus. Data presented are from single experiments with at least 4 replica wells per condition.

We have recently shown that *Candida* spp. induce an IFN-β response in mouse BM-DCs [4]. To test for *in vivo* production of IFNs-I upon systemic *Candida* infections, we infected WT and IFNAR1-deficient mice with Ca by lateral tail vein injection (iv). Both infected mouse genotypes produced similar levels of IFN-α with increasing cytokine levels upon disease progression (Figure 1A). We were unable to detect serum IFN-βlevels under the same experimental conditions, since IFN-β is notoriously known for its low expression levels *in vivo* and thus remained below the detection limit. Nevertheless, BM-DCs from both WT and IFNAR1-deficient mice produced equal levels of IFN-β upon Ca stimulation (Figure S1A). Unlike WT cells, *Ifnar1^−/−^* BM-DCs failed to respond to IFN-β as evident from the absence of STAT1 phosphorylation. (Figure S1B).

To examine a contribution of IFN-I signaling on the infection outcome and fungal dissemination, we compared the survival of WT and *Ifnar1^−/−^* animals infected with various Ca loads. Interestingly, at a dose of 10^5^ Ca colony-forming units (cfus) the lack of IFNAR1 caused a remarkable protection to otherwise lethal infections, which became apparent after one week of injection (Figure 1B). The same phenotype was observed after infection with lower fungal doses of 0.5×10^5^ Ca cfus (Figure S1C), whereas increasing the fungal loads to 5×10^5^ Ca cfus obliterated the protective effect of IFNAR1-deficiency (Figure S1D). Thus, the beneficial effect of lacking an IFN-I response is only evident under infection conditions that do not overburden host protective capacity.

To test whether the improved survival of *Ifnar1^−/−^* mice was a result of increased fungal clearance, we determined fungal burdens in the major organs of infected animals. To avoid premature death of WT mice before sample collection, we infected mice with 0.5×10^5^ Ca cfus. At indicated time points, spleen, liver, brain and kidneys were collected and analysed for the presence of fungal cells by cfu counting. During the first week of infection, Ca cfus remained high only in the kidneys (Figure 1C), which is in agreement with previous findings [24], [25]. In all other organs, fungal burden was controlled by the immune system and either stayed low (brain) or progressively decreased over time (spleen and liver) suggesting successful clearance (Figure S1E). For any organ investigated, we did not observe a significant difference in Ca cfus between WT and *Ifnar1^−/−^* mice. We also investigated the *in vitro* Ca killing capacity of host phagocytes with suspected or known functions in the clearance of fungal infections. Again, we did not observe significant differences in the *in vitro* Ca killing capacities of phagocytes from WT or *Ifnar1^−/−^* mice (Figure 1D). In conclusion, we demonstrate that Ca-induced IFN-I signaling mediates detrimental host effects during disseminated and invasive infections, though not by altering fungal clearance. These data suggest that other mechanisms confer resistance to systemic Ca infections in a host with defective IFN-I signaling.

### IFN-I signaling augments the inflammatory host response to *Candida*

**Figure 2:**
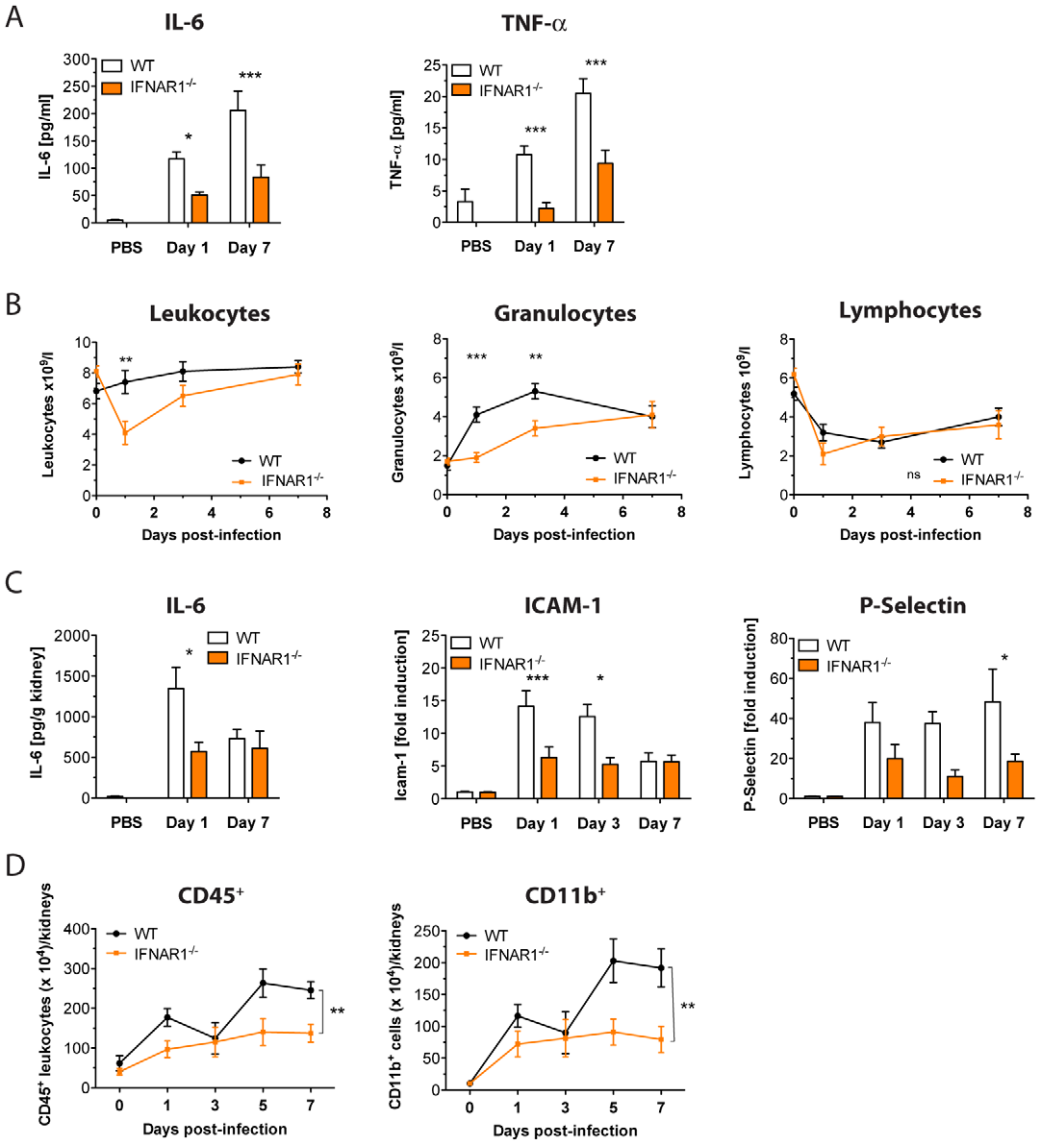
IFN-I signaling promotes hyper-inflammatory immune responses. Mice of the indicated genotype were injected with a lethal dose of 1×10^5^ cfus Ca. At indicated time points, serum/whole blood and kidneys were collected. (A) Sera concentrations of IL-6 and TNF-α were measured using a multiplex bead array system. Data presented show the mean ± SEM of four independent experiments (n = 7–12 mice per group). (B) Blood cell populations were analysed by an automated blood counter. Data presented show the mean ± SEM of two independent experiments (n = 6–8 mice per group). Plotted are the absolute numbers of leukocytes, granulocytes, and lymphocytes expressed as cell number ×10^9^/l. (C) IL-6 concentrations in kidney supernatants were measured using a multiplex bead array system. Gene expression levels of *Icam-1* and *P-Selectin* were quantified by qPCR in kidney total RNA. Data presented show the mean ± SEM of three independent experiments (n = 7–12 mice per group). (D) Kidney leukocytes were enriched and characterized by multi-label flow cytometry. Graphs show absolute numbers of leukocytes (CD45^+^) and myeloid cells (CD11b^+^) per total mouse kidneys. Data presented is one representative of two independent experimental repeats (n = 3–5 mice per group).

We hypothesized that *Ifnar1^−/−^* mice may be more resistant to Ca infections because IFN-I signaling is able to enhance deleterious inflammatory responses. Therefore, we quantified sepsis-relevant inflammatory mediators in serum and kidney, the critical target organ, during the first week of Ca infection. In support of our hypothesis, *Ifnar1^−/−^* mice showed significantly reduced serum levels of inflammatory TNF-α and IL-6 when compared to WT mice (Figure 2A). Furthermore, blood counts revealed considerably lower leukocyte numbers in *Ifnar1^−/−^* mice, indicating an attenuated infection-induced haematopoiesis. In particular, granulocyte counts were significantly lower (Figure 2B), suggesting an impaired or delayed granulopoesis in knock-out animals. By contrast, total lymphocyte numbers did not differ significantly at any time point (Figure 2B). Taken together, these results demonstrate that IFN-I signaling triggers an early systemic release of inflammatory cytokines and contributes to the Ca-induced robust granulopoesis.

As observed for the systemic inflammatory response, early IL-6 levels were significantly reduced at day 1 post infection in kidneys of *Ifnar1^−/−^* mice when compared to WT animals (Figure 2C). We also quantified expression levels of additional inflammation mediators with a known involvement in acute kidney injury [26], including the major adhesion molecules ICAM-1 and P-Selectin. In agreement with attenuated inflammation, we detected reduced renal expression of these adhesins in *Ifnar1^−/−^* mice (Figure 2C). The decrease of these critical cytokines and adhesion molecules in kidneys of knock-out mice strongly suggested a reduced immune cell infiltration in the infected organ. To test this notion, we isolated leukocytes from Ca-infected kidneys and evaluated the total number of immune cells (CD45^+^) and myeloid cells (CD11b^+^). In line with the reduced granulopoesis, *Ifnar1^−/−^* kidneys displayed significantly lower numbers of infiltrating leukocytes, which could be attributed to the selective reduction of myeloid cells within the tissue (Figure 2D). In summary, our results indicate that IFN-I signaling strongly promotes both systemic and acute local inflammatory responses in Ca-infected mice by enhancing the expression of pro-inflammatory mediators, as well as the recruitment of innate immune cells.

### IFNs-I promote kidney injury through unrestrained host responses

**Figure 3:**
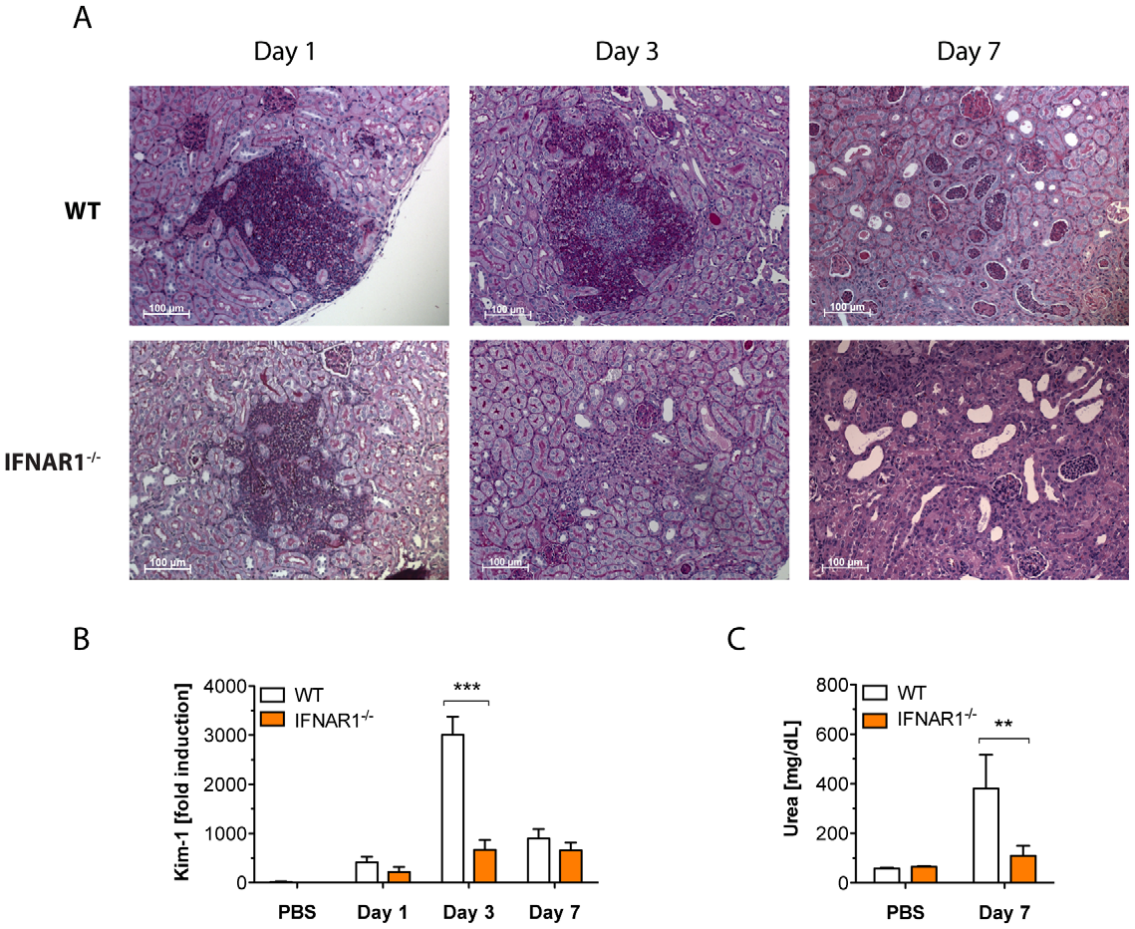
Reduced immunopathology protects *Ifnar1^−/−^* mice from kidney injury. Mice of the indicated genotype were injected with a lethal dose of 1×10^5^ cfus Ca. At indicated time points, serum and kidneys were collected. (A) Histopathology of the cortical part of kidneys at day 1, 3 and 7 post infection. Longitudinal sections of paraffin-embedded organs were stained with periodic acid-Schiff (PAS) to visualize fungal cells. Counterstaining was performed with hematoxylin. (n = 3–4 mice per group) (B) Kidney total RNA was analysed for gene expression of kidney injury marker-1 (*Kim-1*). Data presented show the mean ± SEM of three independent experiments (n = 8–10 mice per group). (C) Urea concentration in serum. Data presented show the mean ± SEM of two independent experiments (n = 7–9 mice per group).

To investigate the pathological consequences of the IFN-I-mediated inflammatory response, we examined the immunohistopathology of infected kidneys from WT and *Ifnar1^−/−^* mice. Histopathological inspection at day 1 after infection revealed typical Ca-containing abscesses in the renal cortex including massive phagocyte infiltrates. There were no obvious differences in the quality and quantity of fungal lesions in WT vs. *Ifnar1^−/−^* mice at this early stage of infection (Figure 3A). However, at day 3, Ca-containing abscesses were still abundantly present in WT mice but were mainly cleared in IFNAR1-deficient animals (Figure 3A). At day 7, the inflammatory process in WT animals spread to the renal tubules and pelvis with extensive tubular cast formation and distortion of the renal architecture. By contrast, there were almost no fresh cellular casts detectable in *Ifnar1^−/−^* mice (Figure 3A). Notably, at that time point, no more Ca cells were detectable within the renal cortex of WT or *Ifnar1^−/−^* mice. Thus, the continuous immune cell recruitment to the renal cortex can be considered an over-reactive host response that may promote kidney pathology.

To test whether the observed damage of renal architecture also impaired kidney function, we measured the expression level of kidney injury molecule-1 (*Kim-1*) in kidneys, as well as urea levels in serum of infected animals. *Kim-1* expression strongly increases in de-differentiated renal proximal tubular epithelial cells upon tissue damage, and is thus a suitable biomarker for early kidney injury [27]. Strikingly, *Kim-1* expression levels peaked at day 3 of infection and were much higher in WT mice when compared to mice lacking IFNAR1 (Figure 3B). The peak of *Kim-1* expression coincided with the presence of Ca lesions in the WT kidney, preceding the subsequent kidney damage, which was evident from the increased urea levels at day 7 (Figure 3C). Taken together, the data let us conclude that IFN-I signaling drives unrestrained inflammatory responses in Ca-infected kidneys, as indicated by the prolonged presence of immune cell infiltrates/fungal abscesses and the abundant formation of cellular casts at later stages of infection. This hyper-inflammatory response increases organ damage as demonstrated by higher expression of *Kim-1* and serum urea levels.

### IFNs-I mediate the recruitment of inflammatory phagocytes to the infected kidney

**Figure 4:**
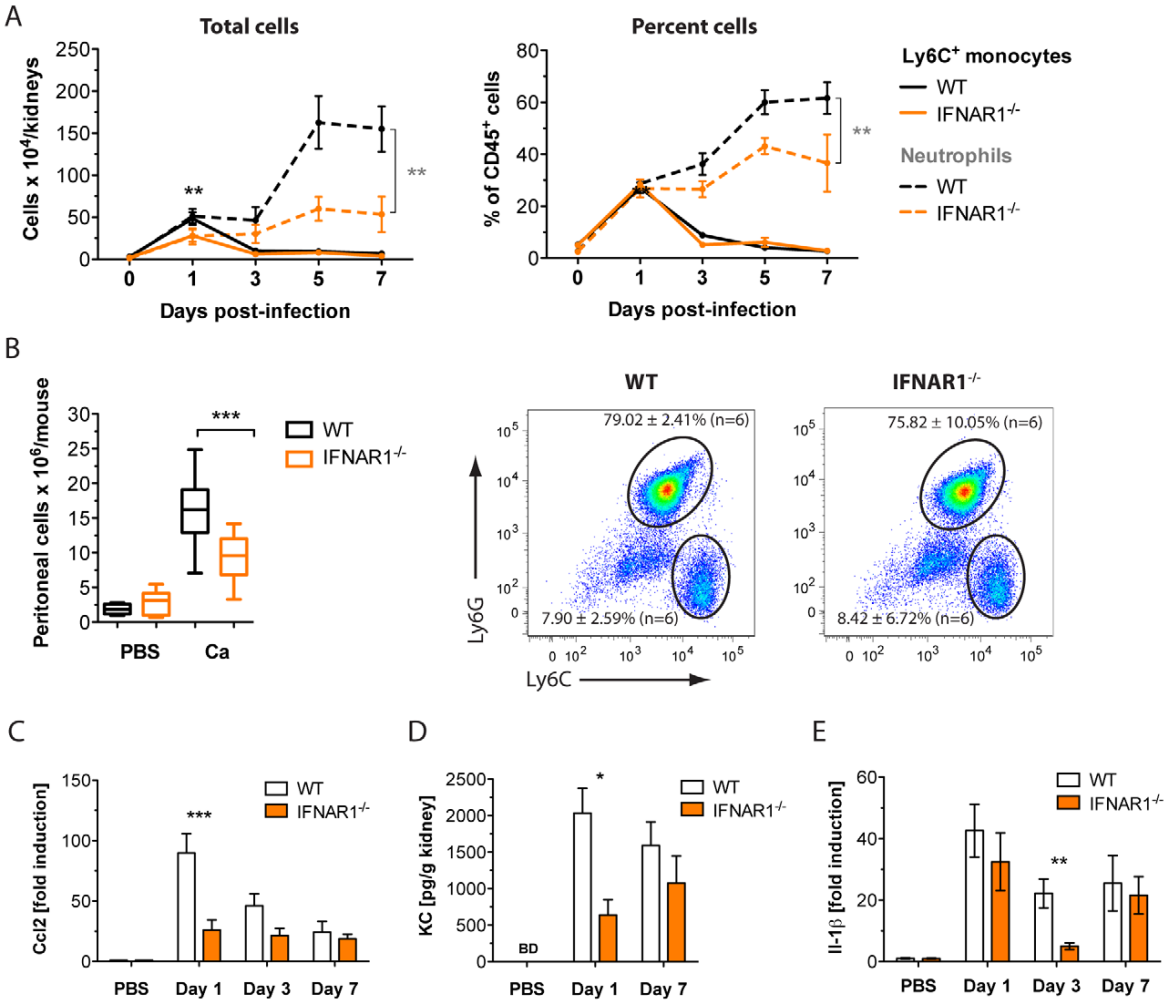
IFN-I signaling promotes inflammatory phagocyte influx to infected sites. (A, C–E) Mice of the indicated genotype were injected with a lethal dose of 1×10^5^ cfus Ca. At indicated time points, kidneys were collected. (A) Kidney leukocytes were enriched and characterized by multi-label flow cytometry. Graphs show absolute numbers of inflammatory monocytes and neutrophils per total mouse kidneys (left) and percent of CD45^+^ cells (right). Data presented is one representative of two independent experimental repeats (n = 3–5 mice per group). (B) Mice of the indicated genotype were ip injected with a sublethal dose of 1×10^7^ cfus Ca. After 4–6 h, peritoneum was flushed and cell number was determined by CASY-counting. Analysis of Ly6G^+^ and Ly6C^+^ peritoneal cells from WT versus *Ifnar1^−/−^* mice (dot plot). (C) Kidney total RNA was analysed for gene expression of *Ccl2*. (D) KC concentrations in kidney supernatants were measured using a multiplex bead array system. Both (C) and (D) show the mean ± SEM of three independent experiments (n = 7–12 mice per group). (E) Kidney total RNA was analysed for gene expression of *Il-1β*. Data presented show the mean ± SEM of three independent experiments (n = 7–12 mice per group).

To identify the cell types contributing to the kidney damage, we thought to determine kidney recruitment kinetics for major immune cell types frequently implicated in inflammatory conditions, including neutrophils [28], inflammatory monocytes [29], [30] and T cells [31], [32]. Therefore, we enriched infiltrating leukocytes from kidneys of Ca-infected WT or *Ifnar1^−/−^* mice to characterize and quantify the different leukocyte populations by flow cytometry, using common immune cell markers (Table S1 in Text S1). As previously observed by others, kidney infections by Ca followed a two-phase innate response [25]. At day 1, neutrophils and inflammatory monocytes accumulated to equal amounts in kidneys of WT mice (Figure 4A). The influx of inflammatory monocytes was transient, peaking at day 1 and declining thereafter. In contrast, neutrophils showed a second wave of massive infiltration starting between day 3 and 5 post infection (Figure 4A). T cells numbers also increased throughout disease, but remained only a minor fraction of the total cell population within the infected kidney (Figure S2A and B). Whereas the recruitment pattern of CD8^+^ and CD4^+^ T cells was comparable between WT and *Ifnar1^−/−^* mice (Figure S2A and B), inflammatory phagocyte infiltrates were significantly less in kidneys of knock-out mice (Figure 4A). Interestingly, *Ifnar1^−/−^* mice displayed significantly lower numbers of inflammatory monocytes and neutrophils during early infection stages and lacked the late massive neutrophil influx (Figure 4A). We further confirmed the lack of neutrophil influx by measuring MPO levels in kidney homogenates. MPO is an oxidative granular enzyme found primarily in neutrophils and is therefore used to quantify tissue neutrophil content [33]. In agreement with reduced neutrophil numbers, MPO levels remained low in *Ifnar1^−/−^* kidneys at day 7 post infection (Figure S2C). We further confirmed the apparent recruitment defect of monocytes and neutrophils using an intraperitoneal (ip) Ca infection model, which is commonly used to assess leukocyte infiltrations to sites of infection [34], [35]. As expected, we observed significantly reduced peritoneal cell infiltrations in IFNAR1-deficient mice (Figure 4B). Since the cell frequencies of peritoneal Ly6C^hi^ monocytes and neutrophils were similar between WT and *Ifnar1^−/−^* mice (Figure 4B), we concluded that the recruitment of both cell types is equally affected by the absence of IFN-I signaling.

Altered expression levels of the respective cell-specific chemokines also reflected the impaired innate cell recruitment to infected kidneys. Coinciding with the peak of inflammatory monocytes, *Ccl2* expression levels were strongly reduced in kidneys of *Ifnar1^−/−^* mice (Figure 4C). Likewise, the early expression of the neutrophil chemokine KC was diminished in knock-out animals (Figure 4D). In contrast, the late neutrophil influx seemed to be independent of KC signaling, since both WT and *Ifnar1^−/−^* mice displayed comparable levels of the chemokine at day 7. Locally produced IL-1β represents another chemo-attractant signal for neutrophils during inflammatory conditions [36]. As expected from the increased neutrophil accumulations, we also detected elevated expression levels of *IL-1β* in kidneys of WT mice (Figure 4E). Increased *IL-1β* levels coincided with the peak of KIM-1 expression and occurred just prior to the second wave of neutrophil influx. Together, these data indicate that IFN-I signaling promotes the early recruitment of neutrophils and inflammatory monocytes into Ca-infected kidneys. Elevated numbers of inflammatory cells during the first days of infection seem to drive a late massive influx of neutrophils, which is absent in *Ifnar1^−/−^* animals.

### IFN-I signaling controls recruitment and activation of inflammatory monocytes

The regulation of inflammatory monocyte trafficking and anti-microbial defense during fungal infections remains unexplored. Emigration of Ly6C^hi^ monocytes from the bone marrow (BM) is known to require signaling via the CCR2 receptor. Notably, the induction of the cognate chemokine ligands such as CCL2, CCL7, and CCL8 are regulated by IFNs-I in certain infectious diseases [22], [23].

**Figure 5:**
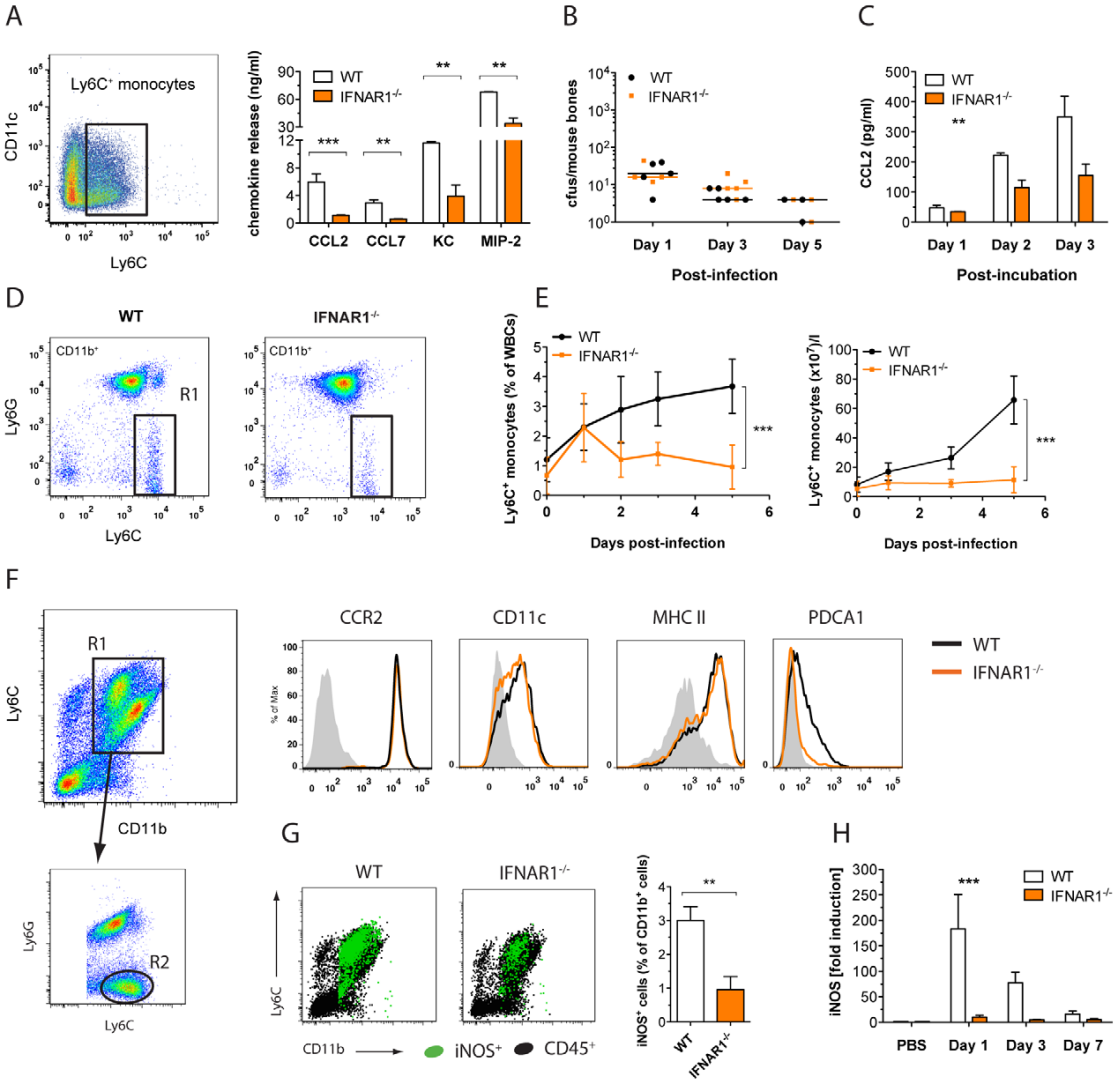
IFN-I signaling regulates Ly6C^hi^ monocytes recruitment and activation. (A) Expression of Ly6C and CD11c on GM-CSF-differentiated BM-DCs at day 8 of culture (dot plot). BM-DCs of the indicated genotypes were stimulated with heat-inactivated Ca. After 24 h, CCL2, CCL7, KC, and MIP-2 release was determined by ELISA or a multiplex bead array system. Data presented show the mean ± SEM of three independent experiments. (B–H) Mice of the indicated genotype were injected with a lethal dose of 1×10^5^ cfus Ca. At indicated time points, blood, BM and kidneys were collected. (B) Ca cfus in BM were determined and expressed as cfus/mouse bones (n = 3–5 mice per group). Each symbol represents one mouse; horizontal bars indicate the calculated median. (C) BM from day 1-infected mice was isolated and placed in culture. At indicated time points, CCL2 release into the media was measured (n = 3 mice per group). (D) Expression of Ly6C and CD11b on blood leukocytes at day 5 of Ca infection in WT versus *Ifnar1^−/−^* mice. Inflammatory monocytes were gated in R1. (E) Quantification of cells in the R1 gate at different time points post infection. Depicted are percent inflammatory monocytes of total WBCs (left) and absolute cell numbers in ×10^7^/l blood (right). (n = 3–5 mice per group) (F–G) Kidney leukocytes were enriched and characterized by multi-label flow cytometry. (F) Expression of Ly6C and CD11b on kidney leukocytes at day 1 of Ca infection. Inflammatory monocytes are gated in R2 (dot plot). Expression of the inflammatory DC surface markers CCR2, CD11c, MHC II, and PDCA1 by cells in the R2 gate. Solid lines, staining of R2 cells of day 1-infected mice; shaded histograms, for CCR2: staining with isotype control antibody, for CD11c and MHCII: staining of CD11c^−^MHCII^−^ neutrophils, for PDCA1: staining of R2 cells of uninfected mice. Data presented are representatives of three independent experimental repeats (total n = 11–14 mice per group). (G) Kidney leukocytes at day one of infection were stained intracellularly for iNOS. Graphs show iNOS^+^ cells (green) overlayed on total CD45^+^ leukocytes (black). Bar diagram shows the quantification of iNOS^+^ cells in percent of total CD11b^+^ cells. Data presented show the mean ± SEM of two independent experiments (n = 8–9 mice per group). (H) Kidney total RNA was analysed for gene expression of iNOS. Data presented show the mean ± SEM of three independent experiments (n = 7–12 mice per group).

Since we had observed a strongly reduced expression of CCL2 in Ca-infected kidneys of *Ifnar1^−/−^* mice, we thought to investigate the IFNAR1-dependent expression of the chemokine in more detail. Monocytes are known as high producers of chemokines, including CCL2 [22]. Therefore, we stimulated GM-CSF-differentiated BM-DC cultures, which contain about one third Ly6C^+^ monocytes (Figure 5A), with heat-inactivated Ca and determined the release of the two major monocyte-attracting chemokines CCL2 and CCL7 [37]. Whereas BM-derived WT Ly6C^+^ monocytes released CCL2 and CCL7 upon Ca challenge, IFNAR1-deficient cells were strongly impaired in their chemokine response (Figure 5A). The release of IFN-β was similar under all conditions, ensuring equal responsiveness of cells (data not shown). We confirmed the IFNAR1-dependent induction of CCL2/CCL7 in WT cells pre-treated with an anti-IFNAR1 blocking antibody prior to Ca stimulation (Figure S3A). As a control, pre-treatment of WT cells with an unspecific isotype IgG did not alter CCL2 production (data not shown).

In line with the kidney chemokine data, we also observed a reduced expression of two neutrophil-attracting chemokines, KC and MIP-2, in Ca-stimulated *Ifnar1^−/−^* BM-DC cultures (Figure 5A), although neither chemokine is a classical IFN-stimulated gene. Interestingly, simply blocking IFNs-I signaling with the anti-IFNAR1 antibody was not sufficient to reduce the release of KC and MIP-2 (Figure S3A), suggesting that the physical presence of a functional IFN-I receptor, rather than its signaling function, cooperates with other pathways for full activation of neutrophil-specific chemokines.

During infections with microbial pathogens, the initial CCL2 production is triggered in the BM, where it mediates inflammatory monocyte egression into the blood stream [4]. Therefore, we examined whether Ca could induce the local production of CCL2 in the BM. To determine whether Ca resides within the BM during infection, we measured cfu counts of infected WT and *Ifnar1^−/−^* mice. In accordance with previous reports [38], we confirmed that fungal cells are present in the BM. As for other organs, the BM fungal burden did not differ between WT and knock-out animals (Figure 5B). To test whether Ca-infected BM releases CCL2, we isolated BM cells from infected WT and *Ifnar1^−/−^* mice at day 1 and cultured them for a total of 3 days. At the time of BM isolation cellular composition and viability of cells were the same between both mouse genotypes (data not shown). Thereafter, we quantified the daily release of CCL2 into the media. Again, we observed a requirement of IFNs-I for full CCL2 production in Ca-infected BM, which became even more evident after longer periods of culture (Figure 5C). Similar results were obtained for the gene expression levels of *Ccl2* and *Ccl7* in infected BM. At day 3 post infection, the induction of monocyte-attracting chemokines was strongly reduced in absence of IFNAR1 signaling (Figure S3B).

We hypothesized that the diminished CCL2/CCL7 production in the BM of *Ifnar1^−/−^* mice may result in a mobilization defect of inflammatory monocytes. Therefore, we determined the number of circulating inflammatory monocytes in the blood of infected WT and *Ifnar1^−/−^* mice. Indeed, we found significantly reduced inflammatory monocyte counts in the blood of IFNAR1-deficient mice (Figure 5D and 5E). In summary, these data establish CCL2/CCL7 as IFN-I-dependent chemokine signals driving the host response during systemic candidiasis. Through regulating the expression of these chemokines in the BM and the kidneys, IFNs-I contribute to the mobilization of inflammatory monocytes into the blood stream and their subsequent migration to the target organ.

In the context of inflammation, Ly6C^hi^ monocytes differentiate into inflammatory DCs within the tissue [39]. Thus, we were interested to test if monocyte-derived inflammatory DCs are also generated during invasive Ca infections. Therefore, we further characterized Ly6C^hi^ kidney monocytes for common inflammatory DC surface and activation markers using flow cytometry. Cells from both WT and *Ifnar1^−/−^* mice expressed high levels of CCR2, the hallmark receptor of inflammatory monocytes, as well as the two major DC markers CD11c and MHCII (Figure 5F). In infected WT kidneys, inflammatory DCs showed upregulated expression of the activation marker PDCA1 (Figure 5F), as well as extensive intracellular iNOS staining (Figure 5G). Strikingly, in the absence of IFN-I signaling, PDCA1 expression and iNOS^+^ cell numbers were significantly reduced. The defect in inducing *iNOS* gene expression was also confirmed by qPCR in infected kidneys of *Ifnar1^−/−^* mice (Figure 5H). We confirmed these results *in vitro* by stimulating GM-CSF-differentiated BM-DCs with heat-inactivated Ca. As for inflammatory DCs in the kidney, we observed a lack of PDCA1 and iNOS expression in the IFNAR1-deficient Ly6C^+^ monocyte population (Figure S3C). Similar results were obtained in WT cultures that had been pre-treated with an anti-IFNAR1 blocking antibody prior to Ca-challenge (Figure S3D). Again, the levels of CD11c or MHC class II were not affected by the absence of IFN-I signaling (Figure S3C and D). All together, we demonstrate that Ly6C^hi^ monocyte differentiate into inflammatory DCs during invasive Ca infections. Whereas the initial differentiation into DCs seems to be independent of IFN-I signaling, our results suggest a novel role for IFNs-I in the functional maturation of inflammatory DCs based on the appearance of specific activation markers.

### Reducing inflammatory myeloid cell numbers protects mice from lethal Ca challenge

**Figure 6:**
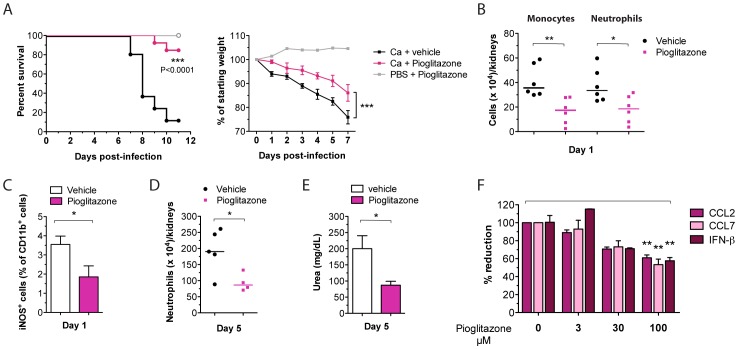
Pioglitazone suppresses lethal inflammatory phagocyte activity. WT mice were injected with a lethal dose of 1×10^5^ cfus Ca and treated daily with 5 mg/kg pioglitazone. (A) Survival and mean percentage of original body weight of drug-treated versus vehicle-treated mice are presented. Data presented show the sum of two independent experimental repeats (n = 13–16 mice per group). (B) Kidney leukocytes were enriched and absolute numbers of inflammatory monocytes and neutrophils determined. Each symbol represents one mouse; horizontal bars indicate the calculated median (n = 6 mice per group). (C) Kidney leukocytes at day 1 of infection were intracellularly stained for iNOS. Bar diagram shows the quantification of iNOS^+^ cells in percent of total CD11b^+^ cells, mean ± SEM (n = 6 mice per group). (D) Absolute numbers of neutrophils in kidneys of treated vs non-treated mice. Each symbol represents one mouse; horizontal bars indicate the calculated median (n = 4–5 mice per group). (E) Urea concentration in serum, mean ± SEM (n = 4–5 mice per group). (F) BM-DCs were pre-treated overnight with indicated concentrations of pioglitazone and stimulated with heat-inactivated Ca the next day. After 24 h of Ca co-incubation, CCL2, CCL7, and IFN-β release were measured by ELISA. Data presented show the mean ± SEM of 3 independent experiments.

IFNs-I promote the inflammation-associated lethal kidney pathology during systemic Ca infections by stimulating the recruitment and activation of Ly6C^hi^ monocytes and neutrophils. However, both cell types are essential for fungal clearance and total elimination renders mice hyper-susceptible to infection [40]. Based on our findings, we hypothesized, that interfering with the activity of these cell types rather than the total elimination would ameliorate host tissue damage and thereby improve the overall outcome of infection. Published evidence suggests that the drug pioglitazone, a synthetic agonist of the nuclear receptor PPAR-γ, impairs inflammatory DC trafficking and associated lung pathology in an influenza mouse model [17]. To test our hypothesis, we treated mice daily with 5 mg/kg pioglitazone or vehicle starting on the day of Ca infection. Strikingly, pioglitazone-treated mice were strongly protected against lethal Ca challenge and experienced a reduced weight loss when compared to non-treated animals (Figure 6A). No significant differences in kidney fungal loads where found between the two mouse groups (data not shown).

To determine whether the protective effect of pioglitazone correlated with an inhibition of inflammatory myeloid cell recruitment, we determined Ly6C^hi^ monocyte and neutrophil numbers in blood and kidneys. In the bloodstream, pioglitazone treatment specifically reduced inflammatory monocyte numbers, while total granulocyte numbers were not affected (Figure S4A). However, in the kidneys, early accumulation of both inflammatory monocytes and neutrophils was equally reduced in drug-treated animals (Figure 6B). In addition to cell recruitment, we investigated the effect of pioglitazone treatment on the activation of inflammatory DCs in infected kidneys. In line with previous reports identifying iNOS as one of the main target genes of PPAR-γ mediated repression [41], we detected significantly reduced iNOS^+^ inflammatory DCs in kidneys of drug-treated mice (Figure 6C), suggesting that pioglitazone also interferes with the functional maturation of inflammatory DCs. The inhibition of iNOS expression could be reproduced *in vitro*, since stimulating pioglitazone pre-treated BM-DCs with heat-inactivated Ca (Figure S4B) led to similar reductions in iNOS expression. Strikingly, the reduced early cell infiltration in kidneys resulted in the absence of the late detrimental neutrophil influx (Figure 6D). Hence, pioglitazone treatment improves kidney damage by diminishing local inflammation, which was evident by reduced serum urea levels at day 5 (Figure 6E). Although there was a general decrease in the magnitude of inflammation in drug-treated animals, the measurements for IL-6 and *Ccl2* in blood and kidney (Figure S4C) did not reach statistical significance due to high variability between mice. Nevertheless, pioglitazone pre-treatment significantly reduced CCL2 and CCL7 release from Ca-stimulated BM-DCs in a dose-dependent manner (Figure 6F), whereas production of KC and MIP-2 was not influenced by the treatment (Figure S4D). A cytotoxic effect of pioglitazone was excluded by live-dead staining of cells after drug treatment (Figure S4E). Interestingly, in addition to monocyte-attracting chemokines, pioglitazone also decreased the initial release of IFN-β by BM-DCs (Figure 6F). This observation prompted us to investigate whether the reduced release of CCL2/CCL7 results from the impaired IFN-β production. Therefore, we pre-treated *Ifnar1^−/−^* BM-DCs with pioglitazone and examined if the remaining release of chemokines from these cells was further decreased by the drug. Pioglitazone treatment was able to even further suppress the production of CCL2 in IFNAR1-deficient cells (Figure S4F). Therefore, the suppressive function on chemokine expression must be a direct effect of the PPAR-γ-mediated transcription inhibition of those genes [42], without involving secondary IFN-β signaling.

**Figure 7:**
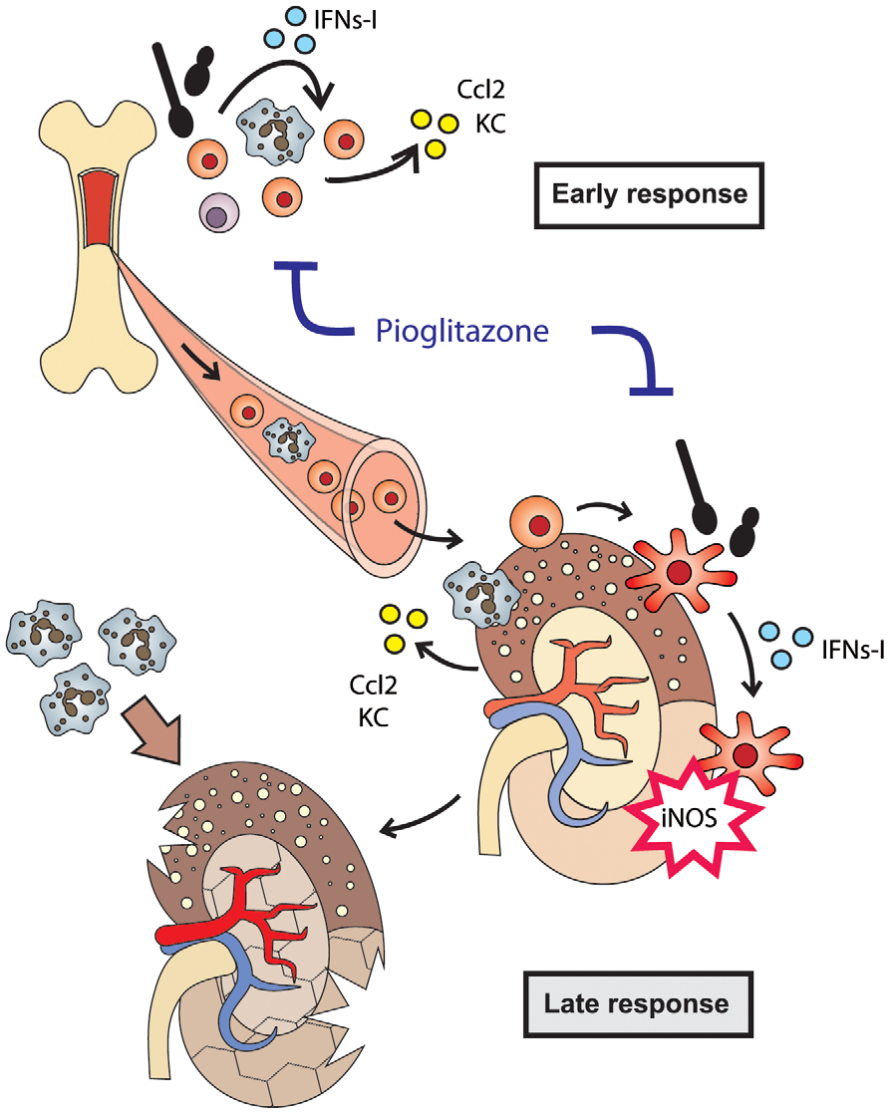
IFNs-I regulate detrimental Ly6C^hi^ monocyte and neutrophil activity. Model of IFN-I-mediated monocyte/neutrophil recruitment and activation of inflammatory DC during invasive Ca infections. Ca recognition triggers an IFN response, which controls the production of various chemokines, including CCL2 and KC, at different anatomical body sites (BM and kidneys). In response to local CCL2 in the BM, Ly6C^hi^ monocytes exit into the blood stream and migrate towards the target organ where they differentiate into inflammatory DCs. To fully functionally mature and become iNOS-producing cells, DCs require signaling through IFNAR1. The high presence and activity of inflammatory DCs and neutrophils in the kidney during the early infection phase promotes a secondary strong influx of neutrophils culminating in lethal immunopathology. The suppressive action of pioglitazone on Ly6C^hi^ monocyte/neutrophil recruitment and function ameliorates hyper-inflammation and kidney pathology.

Taken together, our results suggest that the usage of PPAR-γ agonists might represent a novel therapeutic option to improve survival of a host in the setting of an emerging fungal sepsis. On the basis of our collective data, we therefore propose the following model for the IFN-I-regulated detrimental activity of inflammatory myeloid cells during experimental candidiasis (Figure 7). Recognition of Ca by innate immune cells in the bone marrow initiates the IFN-I–CCL2 cytokine-chemokine cascade and stimulates mobilization of inflammatory monocytes from the bone marrow to the infected organs. Simultaneously, infection-triggered granulopoiesis induces the proliferation and mobilization of neutrophils. Monocytes and neutrophils migrate towards the infected kidneys where CCL2 and KC are produced in an IFNAR1-dependent manner. Inside the renal tissue, inflammatory monocytes acquire a DC-like phenotype through a process not requiring IFN-I signaling. However, the subsequent activation of inflammatory DCs to become high producers of iNOS strictly depends on a functional IFN-I response. High abundance and activity of inflammatory cells causes early tissue damage, leading to the subsequent massive accumulation of neutrophils, whose destructive power ultimately leads to kidney failure. The genetic deficiency of IFNAR1 or the pioglitazone-mediated pharmacological suppression of inflammatory cell recruitment and activation strongly improves Ca-mediated immunopathology and survival of the host.

## Discussion

The present study demonstrates a detrimental effect of IFNs-I during invasive experimental candidiasis. We show that IFN-I signaling stimulates the recruitment of inflammatory innate immune cells, including Ly6C^hi^ monocytes and neutrophils, into the infected kidney, which promote lethal hyper-inflammatory and tissue-damaging immune responses in mice. Importantly, we establish a pharmacological approach by interfering with the activity of those inflammatory cells, which ameliorates disease progression and improves survival of otherwise lethal fungal infections.

In many microbial infections, a disregulated or overshooting immune response rather than the pathogen itself can cause fatal host damage. Likewise, studies comparing host responses to attenuated and virulent Ca strains suggest that fungus-induced inflammation contributes considerably to tissue damage and mortality [16]. The lack of IFN-I signaling in *Ifnar1^−/−^* mice constrains hyper-inflammatory immune reactions and improves survival of mice infected with Ca. Hence, our data support the notion that uncontrolled host responses rather than the fungal growth are a primary cause of death in the murine Candida infection model. Since *Ifnar1^−/−^* mice exhibit similar fungal burdens in critical organs when compared to WT animals, the sepsis resistance phenotype may result from increased host tolerance to the pathogen burden. Therefore, the *Ifnar1^−/−^* mouse model would represent an excellent tool to study virulence factors of Ca, allowing for the mechanistic distinction between tissue damage caused by fungal dissemination versus the hyper-inflammatory host response.

Similar to bacterial infections, IFNs-I can exert both beneficial and detrimental effects during fungal diseases [7], [8]. We have previously reported a role for IFNs-I in supporting persistence of *Candida glabrata* (Cg) within the host. Contrasting with their effects in our Ca infection model, fungal persistence of *Candida glabrata* may arise from an IFN-I-mediated attenuated host response, which facilitates immune evasion by the fungus [4]. This of course implies that specific “patterns of pathogenesis” are exploited by different pathogens, including the use of distinct virulence strategies to generate pathogen-specific contextual cues during infection, which decide about the balance of pro- versus anti-inflammatory functions of IFNs-I. Although a recent study identified *Ifnar1^−/−^* mice as hyper-susceptible to disseminated Ca infections, different fungal strains and infection doses, as well as mouse genetic backgrounds, may explain the discrepancy [5]. Indeed, different microbial loads and injection routes may influence the outcome of infection regardless of the genetic phenotype in the host, which normally determines the capacity of immune surveillance [43], [44].

IFNs-I may dictate a pro- or anti-inflammatory response in host cells depending on the initial IFN-I concentrations targeting responder cells [45], [46]. We report severely reduced levels of inflammatory mediators and innate immune cells in blood and kidneys of *Ifnar1^−/−^* mice upon Ca infection, suggesting a pro-inflammatory role of IFNs-I in this infection model. Notably, IFNs-I have been reported to exert an inhibitory effect on inflammasome activation and the subsequent release of pro-inflammatory IL-1βin mice. The resulting impairment of inflammation-mediated protective immunity renders mice hyper-susceptible to invasive Ca infections [47]. In this mouse model, however, massive IFN-I production was pre-induced with repeated injections of poly-IC prior to fungal infection. We believe that the mechanistic difference to our model is explained by the level of IFNs-I induced between the different challenges, thereby changing the ratio of inflammatory versus anti-inflammatory effector functions. Indeed, in our infection model, where IFNs-I are not produced prior to fungal challenge, we are unable to detect an IFN-I-mediated inhibition of IL-1βproduction. Consistent with our model, we observe unchanged or even lower concentrations of IL-1β in kidney homogenates of *Ifnar1^−/−^* mice.

Invasive Ca infections progress mainly in the kidneys and the consequent organ damage likely contributes to the overall pathology of disease [48]. Thus, we focused on the immune response and pathology of the kidney to further investigate the organ-specific inflammatory response to Ca. Interestingly, we show a significantly reduced early accumulation of myeloid cells, including inflammatory monocytes and neutrophils, and a lack of the second massive neutrophil infiltration in kidneys of IFNAR1-deficient animals. While IFN-I signaling seems to play a general role in promoting monocyte recruitment, as evident from other studies [19], [22], [49], their effects on neutrophil trafficking and function are quite divergent between different infection models [12], [13], [50]. Similar to our Ca infection model, IFNs-I mediate the recruitment of both inflammatory monocytes and neutrophils into kidneys during experimental glomerulonephritis [51]. Also in this model, the consequent higher abundance of myeloid cells worsens disease pathology, indicating the detrimental role of monocytes and neutrophils in kidney function. In future studies it will be interesting to delineate the individual contributions of monocytes and neutrophils to Ca-induced kidney immunopathology by adopting antibody-mediated depletion strategies. One may speculate that early tissue damage caused by the activity of inflammatory innate cells triggers the second wave of neutrophil influx in our infection model. There is evidence that danger signals generated through cellular damage at the site of inflammation attract neutrophils. Many of these danger signaling pathways converge on IL-1β as a key orchestrator of inflammation and cell recruitment [36]. Indeed, we detect higher IL-1β expression levels in kidneys of WT animals when compared to *Ifnar1^−/−^* mice.

Likewise neutrophils, inflammatory monocytes/DCs have been implicated in the immunopathology of various diseases, including infectious or autoimmune diseases [52]. In other fungal infection models, including respiratory aspergillosis and cryptococcosis, monocytes contribute to the instruction of protective T cell immunity and Th cell differentiation [53], [54]. Inflammatory monocytes emigrate from the BM through CCR2 receptor-mediated signaling and accumulate at the sites of infection [37], [55]. We show here that IFNs-I control the recruitment of Ly6C^hi^ monocytes by inducing CCL2/CCL7 expression both in the BM and the target organ, strongly increasing inflammatory monocyte counts in blood and kidneys of Ca-infected WT mice. Bone marrow mesenchymal stem and progenitor cells are the initial producers of CCL2 and inducers of monocyte emigration during infections [56]. Notably, during infections with *Listeria monocytogenes*, IFNAR1 does not contribute to the early monocyte emigration from the BM within the first 24 hours [57]. Our data confirm the early IFNAR1-independent induction of monocyte-attracting chemokines in the BM during the early response. However, at later stages of infection, IFNs-I become central mediators of CCL2/CCL7 production and monocyte egression. Hence, the IFN-I-driven CCL2/CCR2 pathway is of general importance for controlling inflammatory monocyte recruitment during microbial infectious diseases.

Due to a defect of monocyte egression from the BM, mice lacking CCL2 or the cognate receptor CCR2 retain most monocytes in the BM [37], [55]. In contrast to *Ccr2^−/−^* or *Ccl2^−/−^* mice, *Ifnar1^−/−^* mice do not show this retention phenotype, most likely because IFN-I signaling might also affect the primary differentiation or proliferation of monocytes within the BM [58]. Upon arrival in the infected tissue, Ly6C^hi^ monocytes further differentiate into inflammatory DCs to execute their anti-microbial defense [21], [55]. In addition to the impaired recruitment of inflammatory monocytes, *Ifnar1^−/−^* mice show defects in the activation of inflammatory DCs, as evident by their reduced PDCA1 and iNOS expression. Whereas iNOS is a well-established functional marker of inflammatory DCs, a PDCA1^+^ inflammatory-like DC subset has been only recently associated with higher expression of pro-inflammatory cytokines and increased T cell-stimulatory potential [59]. IFNs-I have been known for their capacity to differentiate human blood monocytes *in vitro* into a special DC subset, termed IFN-DCs [60]. Interestingly, the ability of IFNs-I to tip the balance in monocyte terminal differentiation has been also noted during chronic inflammation [19], where IFNs-I sustain the inflammatory conditions by inhibiting the terminal differentiation of monocytes into anti-inflammatory tissue macrophages, perhaps by promoting their differentiation into inflammatory DCs. Since inflammatory monocytes seem to carry out several central functions in anti-fungal immunity, including propagation of inflammation and T cell instruction, it will be of particular interest to establish a possible role of these cells during fungal diseases in the patient setting, including the search for genetic polymorphisms in CCR2 or CCL2 that might modulate the outcome of fungal infections. Notably, SNPs in STAT1, a key mediator of both IFN-I and IFN-II (IFN-γ) signaling, are implicated in the outcome of invasive fungal infections in humans [61].

Here, we show that pharmacological suppression of monocytes and neutrophils as achieved by treating mice with pioglitazone, a synthetic agonist of the nuclear receptor PPAR-γ, can rescue animals from Ca-mediated immunopathology. Stimulating PPAR-γ antagonizes inflammatory responses by repressing the transcriptional activation of NFκB target genes, including CCL2, TNF-α and iNOS [62], [63]. Interestingly, a prophylactic pioglitazone treatment has been recently adopted to reduce detrimental inflammatory DC infiltration into the lungs during influenza virus infections [17]. Strikingly, we demonstrate here that pioglitazone treatment of Ca-infected mice reduces the early accumulation of inflammatory monocytes, as well as neutrophils in kidneys. Furthermore, treatment also strongly impairs the neutrophil influx at later stages of infection. The drug also suppresses the activation of inflammatory DCs, thereby diminishing organ inflammation. Thus, pioglitazone treatment precisely phenocopies the observations in *Ifnar1^−/−^* mice, including the reduced inflammatory cell recruitment and functional maturation of inflammatory DCs. Activation of individual nuclear receptors is believed to repress specific subsets of inflammatory target genes with different functions, resulting in distinct biological consequences for the host response [64]. Notably, our data indicate that pioglitazone represses a specific gene subset *in vivo*, which seems to overlap with the IFN-I target genes driving the lethal inflammation during invasive candidiasis. Previous studies have revealed that pioglitazone treatment rescues mice from lethal influenza virus infections and reduces disease activity of septic peritonitis or murine lupus [17], [62], [65]. In support of the beneficial effects of pioglitazone, we show that treatment of Ca-infected mice ameliorates renal immunopathology and improves animal survival.

Our data suggest that the modulation of Ly6C^hi^ monocyte and neutrophil numbers, as observed for *Ifnar1^−/−^* and pioglitazone-treated mice, has beneficial effects for the host by dampening the hyper-inflammation. The detrimental immunopathology most likely results from the biphasic recruitment and activation of inflammatory monocytes and neutrophils, both of which contribute to the fatal kidney pathology. In support of our hypothesis, depletion of Gr1^+^ cells at day 7 of Ca infection, which removes both monocytes and neutrophils, increases survival of mice [40].

Taken together, our work identifies IFN-I signaling as a central mediator of inflammatory innate immune cell migration into infected organs, demonstrating a pivotal yet detrimental role for IFNs-I in fungal pathogenesis *in vivo*. The inflammatory cascade driven by the sustained expression of IFN-I-regulated inflammatory genes substantially contributes to tissue damage observed in infected mice. The pharmacological suppression of inflammatory monocytes and neutrophils shows that interfering with those cell types during invasive Ca infections improve immunopathology and disease outcome. Thus, this work expands the spectrum of detrimental inflammatory immune cells during Ca infections beyond neutrophils. Our study provides a novel mechanism for the role of IFNs-I in sepsis progression, coupling IFN-I target genes such as CCL2 and iNOS to the recruitment and activation of inflammatory monocytes/DCs with considerable host-destructive potential. The direct connection between IFNs-I and inflammatory monocytes might be of general importance in microbial diseases where an IFN-I response is detrimental for the host. We propose that the beneficial effects of PPAR-γ agonists may also apply to other infectious diseases where inflammatory myeloid cells promote tissue damage, including parasitic infections or tuberculosis [18], [66].

## Materials and Methods

### Ethics statement

All animal experiments were discussed and approved through the University of Veterinary Medicine Vienna institutional ethics committee and carried out in accordance with animal experimentation protocols approved by the Austrian law (GZ 680 205/67-BrGt/2003, GZ-BMWF-68.205/0233-II/10b/2009 and GZ-BMWF-68.205/0231-II/3b/2011).

### Fungal strains and growth conditions

The *Candida albicans* (Ca) strain used in this study was the standard clinical isolate SC5314 [67]. Fungal cells were grown to the logarithmic growth phase in single-use, pyrogen- and endotoxin-free sterile flasks. For detailed information see supplemental text and materials.

### Mouse models and pioglitazone treatment

*Ifnar1^−/−^* mice on C57BL/6 background [68] were bred at Biomodels Austria, University of Veterinary Medicine. C57BL/6 wild type controls were purchased from Charles River. Mice were housed under specific pathogen-free conditions according to FELASA guidelines. Male mice were challenged on day 0 via the lateral tail vein with 1×10^5^ Ca colony-forming units (cfus) per 21 g body weight, if not otherwise stated. Fungal load was adjusted to individual mouse weights. For survival experiments, mice were monitored for 14–35 days. Groups of mice were sacrificed on different days post infection (p.i.) for analysis of macroscopic and histological changes, fungal organ burden, and changes in the levels of cytokines and other inflammatory mediators in blood as well as organ homogenates. Mice were treated daily with 5 mg/kg pioglitazone in 0.5% methylcellulose/PBS via ip injections starting on day 0 with the Ca infections. The control mice received vehicle only. Weight loss was monitored every other day as a measure of morbidity.

### Determination of fungal burden

Mice were sacrificed and spleen, liver, kidneys, and brain were removed aseptically at necropsy, rinsed with sterile PBS, weighted, and placed in 1.5 ml sterile tissue lysis buffer (200 mM NaCl, 5 mM EDTA, 10 mM Tris, 10% glycerol, 1× protease inhibitor cocktail (Roche)) on ice. The organs were aseptically homogenized using an Ika T10 basic Ultra-Turrax homogenizer (Ika, Staufen). Serial dilutions of homogenates were plated in triplicate on YPD (1% yeast extract, 1% peptone, 2% dextrose) plates containing ampicillin, tetracycline, and chloramphenicol. Colonies were counted after 48 h of incubation at 30°C. The fungal burden was calculated as cfus per gram of tissue.

### Clinical parameters, haematology and histopathology

To assess kidney tissue damage, levels of blood urea (blood urea nitrogen - BUN) were determined in serum samples by a routine veterinarian diagnostics laboratory (InVitro GmbH). For haematology, blood samples were collected in K-EDTA-coated tubes (Sarstedt) and analysed with an automated blood counter (V-Sight, A. Menarini). For histology, parts of organs were fixed with buffered 4% paraformaldehyde, and paraffin-embedded sections were stained with hematoxylin-eosin (HE) or periodic acid-Schiff (PAS) stain according to standard protocols.

### Cytokine measurements by ELISA

The amount of IFN-β released in cell culture supernatants was assayed using the Verikine mouse IFN-β ELISA kit (R&D systems). Serum IFN-α was measured using the Luminex system with Procarta Cytokine Profiling Kits; TNF-α and IL-6 in serum were determined using the Mouse CBA flex sets (BD Biosciences); CCL7, KC, and MIP-2 using Procarta Immunoassays (Panomics-Affymetrix); CCL2 using a commercial ELISA Set (Biolegend); all according to the manufacturer's instructions.

For cytokine quantification in tissues, organ homogenates of Ca-infected mice prepared as described for fungal burden determination were centrifuged twice (1,500× g, 15 min, 4°C) and diluted prior to measurement. Myeloperoxidase (MPO) was determined by the Mouse MPO ELISA kit (Hycult Biotechnology); IL-6 and KC chemokine were measured using the Mouse CBA flex sets (BD Biosciences); all conditions were according to the manufacturer's instructions.

### Flow-cytometry analysis of immune cells

Blood preparation and leukocyte enrichment from kidneys were performed as described in the supplemental material. Blood and kidney leukocytes were stained with the appropriate combination of FITC-labeled anti-Ly6G (1A8, Biolegend) or anti-CD3 (145-2C11, BD Biosciences), PE-labeled anti-PDCA1 (eBio129c, eBioscience), anti-CD4 (RM4-5, BD Biosciences), APC-Cy7-labeled anti-CD45 (30-F11, BD Biosciences), PerCP-Cy5.5-labeled anti-CD11c (N418, Biolegend), Pacific Blue-labeled anti-Ly6C (HK1.4, Biolegend), BD Horizon-labeled CD11b (M1/70; BD Biosciences) or anti-CD8 (53-6.7, BD Biosciences), after blocking of Fc receptors with anti-CD32/CD16 (93, eBioscience).

Intracellular staining of iNOS was performed with anti-NOS2 (M-19, Santa Cruz) and DyLight 649-conjugated anti-rabbit IgG (Jackson Immuno Research) according to the application guidelines of BD Bioscience. Anti-CCR2 (MC-21) was kindly provided by Matthias Mack. Data were acquired using a FACSAria (BD Biosciences) and analysed using the FlowJo software (Tree Star). Leukocyte characterization was performed on gated CD45^+^ cells. Leukocytes were further identified according to cell-specific markers as listed in Table S1 in Text S1.

### Innate immune cell isolation and Candida killing assay

For the preparation of exudate neutrophils and peritoneal macrophages (Mphs), C57BL/6 mice were ip-injected with 0,5 ml 10% proteose peptone (Sigma, St. Louis, MO, USA)/PBS. After 4 h (for neutrophils) or 3 days (for peritoneal macrophages), the peritoneum was flushed with 7 ml PBS containing 50 U/ml heparin to collect cells. For isolation of resting Mphs, peritoneum of untreated mice was flushed. For isolation of bone marrow resident neutrophils, bone marrow was collected from mice. After lysis of red blood cells, samples were separated on a discontinuous Percoll gradient: from bottom to top 78%, 69%, and 52% Percoll (GE Healthcare). Gradient was centrifuged at 1500×g and 4°C for 30 min. Cells accumulating in the interphase between the 78% and 69% Percoll layer were collected as neutrophils. All different types of immune cells were plated in RPMI supplemented with 10% heat-inactivated FCS and used for co-culture with fungi.

For the *in vitro* Ca killing assay, innate immune cells were plated in replicates at a density of 1×10^5^ cells/well of 96-well plates. Cells were incubated with Ca at indicated MOIs and for indicated time. After incubation, mammalian cells were lysed by addition of Triton X 100 to a final concentration of 1%. After lysis, wells were extensively scrapped, 2× washed with PBS and surviving Ca was determined by plating serial dilutions of the collected media and washes in duplicates on YPD plates containing ampicillin (Sigma). The percentage of killing was calculated according to the following formulas (df = dilution factor):
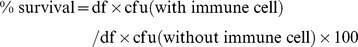


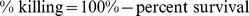



### Intraperitoneal leukocyte recruitment

Male mice were injected intraperitoneally with 1×10^7^ Ca colony-forming units (cfus). After 4–6 hours, peritoneal cells were collected with sterile PBS, and the total cell number was assessed in a CASY counter. Cells were stained for flow-cytometry analysis with FITC-labeled anti-Ly6G (1A8, Biolegend), Pacific Blue-labeled anti-Ly6C (HK1.4, Biolegend), and BD Horizon-labeled CD11b (M1/70; BD Biosciences).

### Reverse transcription and real-time PCR analysis

RNA sample preparation, reverse transcription and real-time PCR were performed as described in the supplemental material. Relative quantification was performed with the ΔΔCt-method. Expression level of the genes of interest were normalised to the expression level of the housekeeping gene *HPRT*. Real-time PCR data are expressed as fold increase of mRNA expression over baseline levels (uninfected mice). All primers used in this study are listed in Table S2 in Text S1.

### Statistical analysis

Statistical analysis of data was performed using the Prism graphing and analysis software (Graphpad). Survival data were compared using the logrank test. Candida cfu data were analysed using the non-parametric Mann-Whitney-test. Time-kinetic comparisons of WT and *Ifnar1^−/−^* mice data at every time point were performed using Two-way ANOVA followed by a Bonferroni post-test. Two-group comparisons were done with the Student's t test. In all cases, *P*<0.05 was considered significant. *p<0.05; **p<0.01; ***p<0.001; ns, not significantly different.

### Gene IDs

Bst2 (PDCA1): ENSMUSG00000046718, Ccl2: ENSMUSG00000035385, Ccl7: ENSMUSG00000035373, Ccr2: ENSMUSG00000049103, Cd4: ENSMUSG00000023274, Cd8a: ENSMUSG00000053977, Cxcl1 (KC): ENSMUSG00000029380, H2-D1 (MHC II): ENSMUSG00000073411, Havcr1 (KIM-1): ENSMUSG00000040405, Icam1: ENSMUSG00000037405, Ifna2: ENSMUSG00000078354, Ifnar1: ENSMUSG00000022967, Ifnb1: ENSMUSG00000048806, Il1b: ENSMUSG00000027398, Il6: ENSMUSG00000025746, Itgam (CD11b): ENSMUSG00000030786, Itgax (CD11c): ENSMUSG00000030789, Ly6c1: ENSMUSG00000079018, Mapk14 (p38): ENSMUSG00000053436, Mpo: ENSMUSG00000009350, Nos2 (iNOS): ENSMUSG00000020826, Ptprc (CD45): ENSMUSG00000026395, Selp (P-Selectin): ENSMUSG00000026580, Stat1: ENSMUSG00000026104, Tnf: ENSMUSG00000024401

## Supporting Information

Figure S1 **The role of IFNs-I during experimental candidiasis.** (A) IFN-β release of WT or *Ifnar1^−/−^* BM-DCs stimulated with Ca for 24 h. Data presented show the mean ± SEM of 4 independent experiments. (B) Phosphorylated STAT1 of lysates from WT or *Ifnar1^−/−^* BM-DCs stimulated with Ca for 2 h. Data presented is one representative of three independent experimental repeats. Mice of the indicated genotype were iv injected with a low dose of 0.5×10^5^ cfus Ca (C) or a high dose of 5×10^5^ cfus (D) and survival was monitored for a period of 21 days. The data here are presented as Kaplan-Meier survival curves and are from one experiment with a total number of 6 or 12 mice per group, respectively. (E) Mice were injected with 0.5×10^5^ cfus Ca. At indicated time points, Ca cfus in brain, spleen, and liver were determined and expressed as cfus/g organ (n = 3 mice per group). Each symbol represents one mouse; horizontal bars indicate the calculated median.
(TIF)

Figure S2 **Immune cell recruitment to kidneys.** Mice of the indicated genotype were injected with a lethal dose of 1×10^5^ cfus Ca. At indicated time points, both kidneys were collected. Kidney leukocytes were enriched and immune cell populations characterized by multi-label flow cytometry. Graphs show CD8^+^ T cells (A) and CD4^+^ T cells (B) as absolute numbers per mouse kidneys (left panels) or as percentage of CD45^+^ cells (right panels). Data presented show the mean ± SEM of one experiment with 5 mice per time point. (C) MPO concentrations in kidney supernatants were measured by ELISA. Data presented show the mean ± SEM of four independent experiments (n = 8–12 mice per group).
(TIF)

Figure S3 **Recruitment and activation of inflammatory monocytes requires IFN-I signalling.** (A) WT BM-DCs were pre-treated with either an α-IFNAR1 blocking antibody or an unspecific isotype control prior to stimulation with heat-inactivated Ca. After 24 h, CCL2 CCL7, KC, and MIP-2 release was determined by ELISA or a multiplex bead array system. Data presented show the mean ± SEM of three independent experiments. (B) Mice of the indicated genotype were injected with a lethal dose of 1×10^5^ cfus Ca. At indicated time points, BM was collected and total RNA analysed for gene expression of *Ccl2* and *Ccl7*. Data presented show the mean ± SEM (n = 4–5 mice per group). (C) BM-DCs of the indicated genotypes or (D) WT BM-DCs pre-treated with either an α-IFNAR1 blocking antibody or an unspecific isotype control were stimulated for 24 h with heat-inactivated Ca. Cells were stained for the inflammatory DC markers iNOS, PDCA1, CD11c, and MHCII. For analysis and histogram presentation only Ly6C^+^ cells have been gated. Solid lines; staining of Ly6C^+^ cells after Ca stimulation; shaded histograms; staining of unstimulated culture. Data presented show representatives of two independent experimental repeats.
(TIF)

Figure S4 **Pioglitazone attenuates inflammatory host responses.** (A,C) WT mice were injected with a lethal dose of 1×10^5^ cfus Ca and treated daily with 5 mg/kg pioglitazone. At indicated time points, blood and kidneys were collected. (A) Blood samples were analyzed for the percentage of inflammatory monocytes (left) or granulocytes (right) in total WBCs. Data presented show the mean ± SD of one representative experiment out of two independent repeats (n = 3–5 mice per group). (B) BM-DCs were pre-treated with 100 µM pioglitazone overnight and stimulated the next day with heat-inactivated Ca for 24 h. Expression of iNOS was determined by intracellular staining. For analysis and histogram presentation only Ly6C^+^ cells have been gated. Solid lines; staining of Ly6C^+^ cells after Ca stimulation; shaded histograms; staining of unstimulated culture. Bar diagram shows the quantification of iNOS^+^ cells of total Ly6C^+^ cells, mean ± SD (n = 4). (C) Sera concentrations of IL-6 (left) were measured using ELISA. Kidney total RNA was analysed for gene expression of *Ccl2*. Data presented show the mean ± SEM (n = 5–6 mice per group). (D) BM-DCs were pre-treated overnight with indicated concentrations of pioglitazone and stimulated with heat-inactivated Ca the next day. After 24 h of Ca co-incubation, KC and MIP-2 release were measured by a multiplex bead array system. Data presented show the mean ± SEM of 3 independent experiments. (E) Cytotoxic effect of pioglitazone. BM-DCs were pre-treated with varying concentrations of pioglitazone for 24 h and cell viability was determined by live-dead staining of cells. Data presented shows one representative of two independent experimental repeats. (F) WT or *Ifnar1^−/−^* BM-DCs were pre-treated with 100 µM pioglitazone overnight and stimulated the next day with heat-inactivated Ca. After 24 h, CCL2 release was measured by ELISA. Data presented show the mean ± SD of 3 independent experiments.
(TIF)

Text S1 **Supplemental information, including 4 Figures as well as 2 Tables and additional experimental procedures.**
(DOC)
