# Supplementary figures and images for: Type I Interferons Promote Fatal Immunopathology by Regulating Inflammatory Monocytes and Neutrophils during Candida Infections
Source: PLoS Pathog. 2012 Jul 26;8(7):e1002811. doi: 10.1371/journal.ppat.1002811 (PMC3406095; doi:10.1371/journal.ppat.1002811)

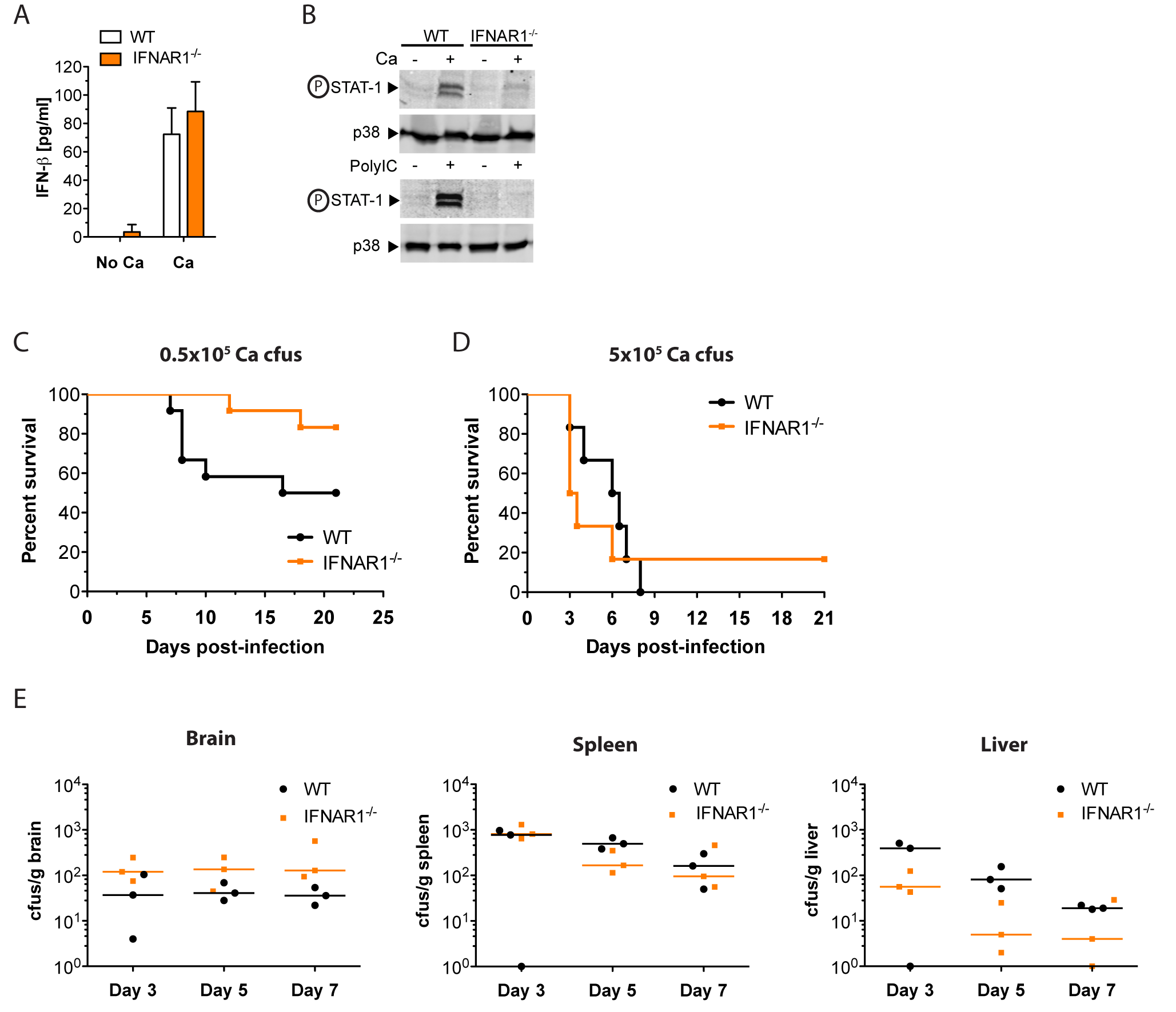

Supplement: Figure S1 — The role of IFNs-I during experimental candidiasis. (A) IFN-β release of WT or Ifnar1−/− BM-DCs stimulated with Ca for 24 h. Data presented show the mean ± SEM of 4 independent experiments. (B) Phosphorylated STAT1 of lysates from WT or Ifnar1−/− BM-DCs stimulated with Ca for 2 h. Data presented is one representative of three independent experimental repeats. Mice of the indicated genotype were iv injected with a low dose of 0.5×10^5^ cfus Ca (C) or a high dose of 5×10^5^ cfus (D) and survival was monitored for a period of 21 days. The data here are presented as Kaplan-Meier survival curves and are from one experiment with a total number of 6 or 12 mice per group, respectively. (E) Mice were injected with 0.5×10^5^ cfus Ca. At indicated time points, Ca cfus in brain, spleen, and liver were determined and expressed as cfus/g organ (n = 3 mice per group). Each symbol represents one mouse; horizontal bars indicate the calculated median. (TIF) [file ppat.1002811.s001.tif]

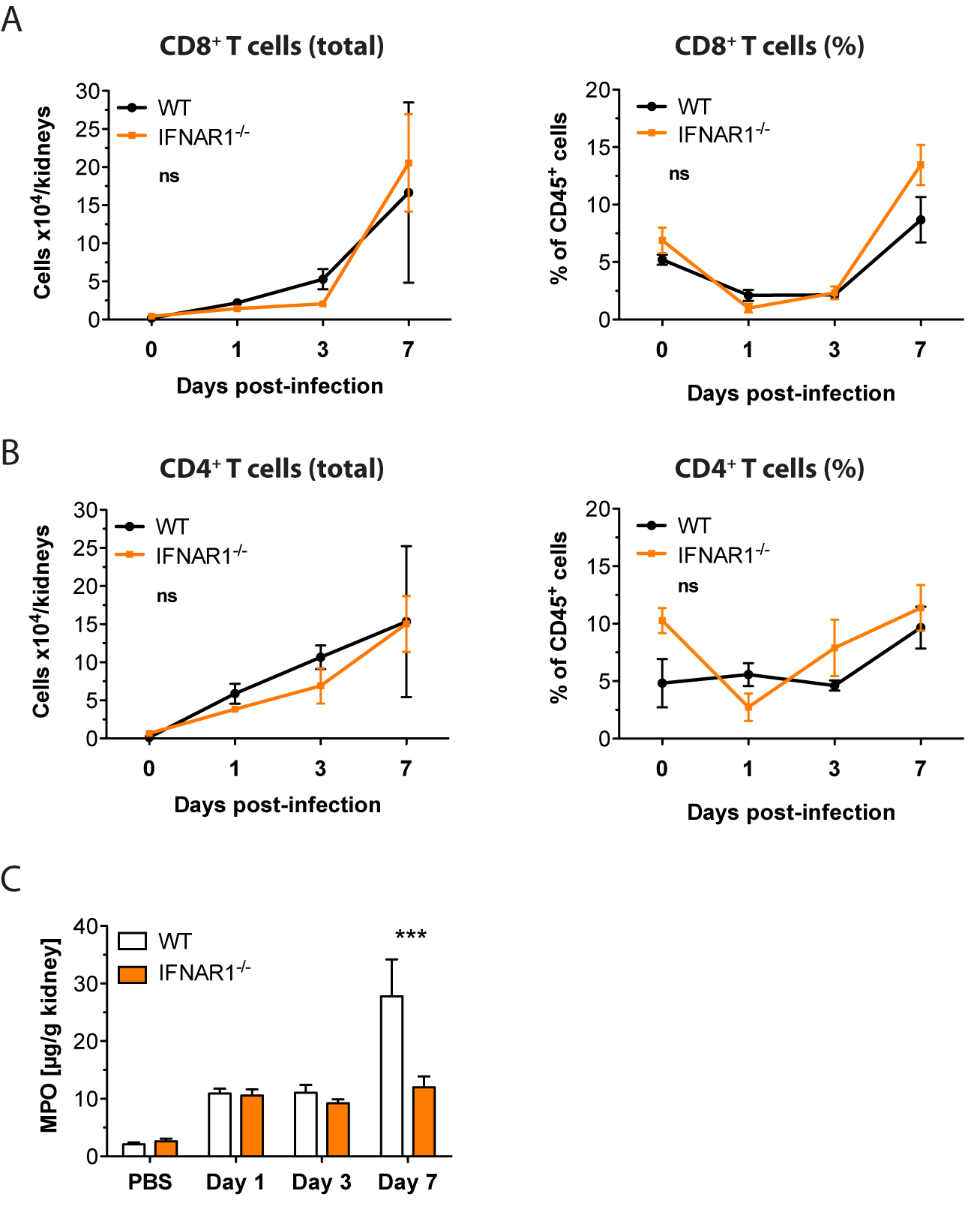

Supplement: Figure S2 — Immune cell recruitment to kidneys. Mice of the indicated genotype were injected with a lethal dose of 1×10^5^ cfus Ca. At indicated time points, both kidneys were collected. Kidney leukocytes were enriched and immune cell populations characterized by multi-label flow cytometry. Graphs show CD8+ T cells (A) and CD4+ T cells (B) as absolute numbers per mouse kidneys (left panels) or as percentage of CD45+ cells (right panels). Data presented show the mean ± SEM of one experiment with 5 mice per time point. (C) MPO concentrations in kidney supernatants were measured by ELISA. Data presented show the mean ± SEM of four independent experiments (n = 8–12 mice per group). (TIF) [file ppat.1002811.s002.tif]

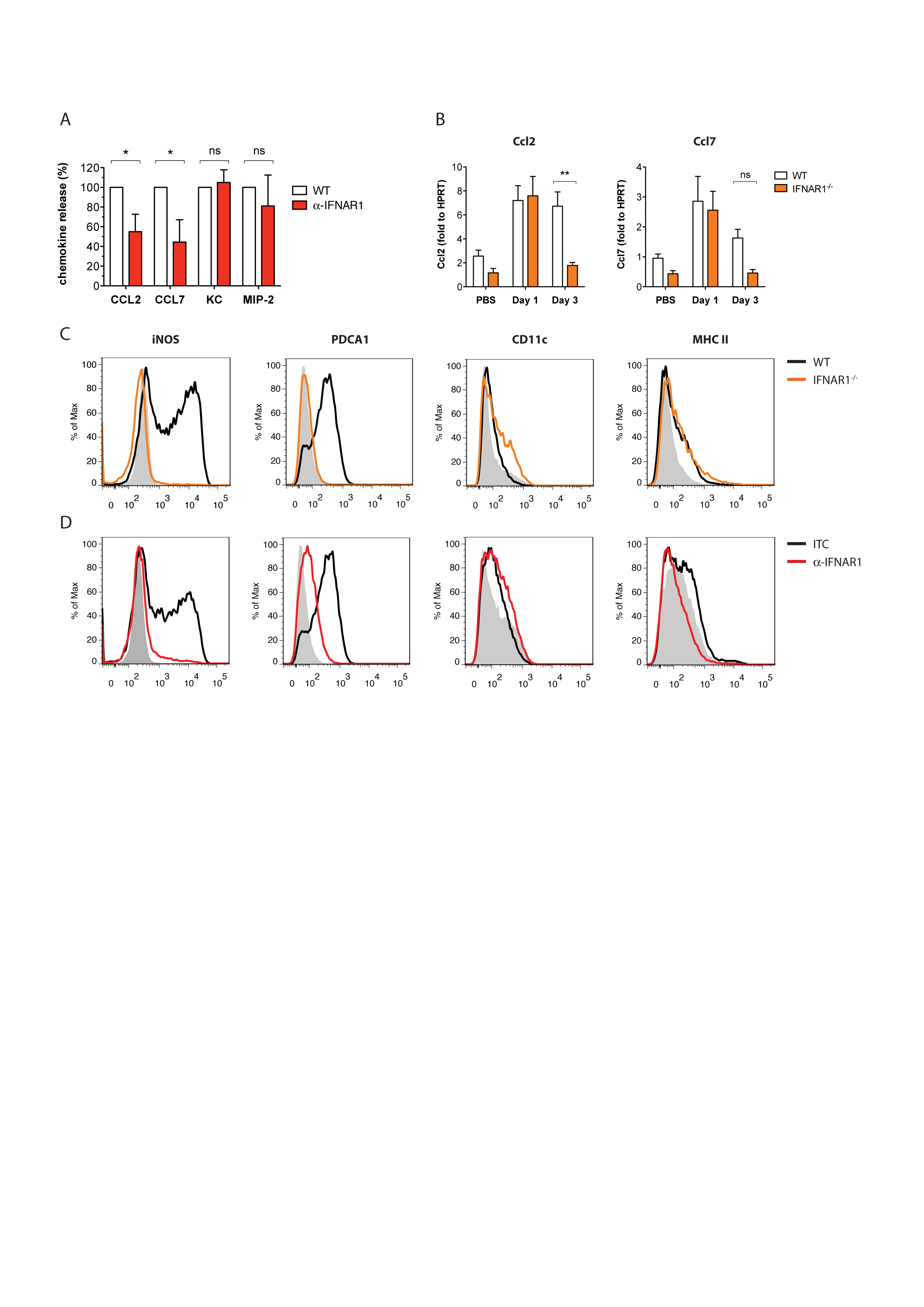

Supplement: Figure S3 — Recruitment and activation of inflammatory monocytes requires IFN-I signalling. (A) WT BM-DCs were pre-treated with either an α-IFNAR1 blocking antibody or an unspecific isotype control prior to stimulation with heat-inactivated Ca. After 24 h, CCL2 CCL7, KC, and MIP-2 release was determined by ELISA or a multiplex bead array system. Data presented show the mean ± SEM of three independent experiments. (B) Mice of the indicated genotype were injected with a lethal dose of 1×10^5^ cfus Ca. At indicated time points, BM was collected and total RNA analysed for gene expression of Ccl2 and Ccl7. Data presented show the mean ± SEM (n = 4–5 mice per group). (C) BM-DCs of the indicated genotypes or (D) WT BM-DCs pre-treated with either an α-IFNAR1 blocking antibody or an unspecific isotype control were stimulated for 24 h with heat-inactivated Ca. Cells were stained for the inflammatory DC markers iNOS, PDCA1, CD11c, and MHCII. For analysis and histogram presentation only Ly6C+ cells have been gated. Solid lines; staining of Ly6C+ cells after Ca stimulation; shaded histograms; staining of unstimulated culture. Data presented show representatives of two independent experimental repeats. (TIF) [file ppat.1002811.s003.tif]

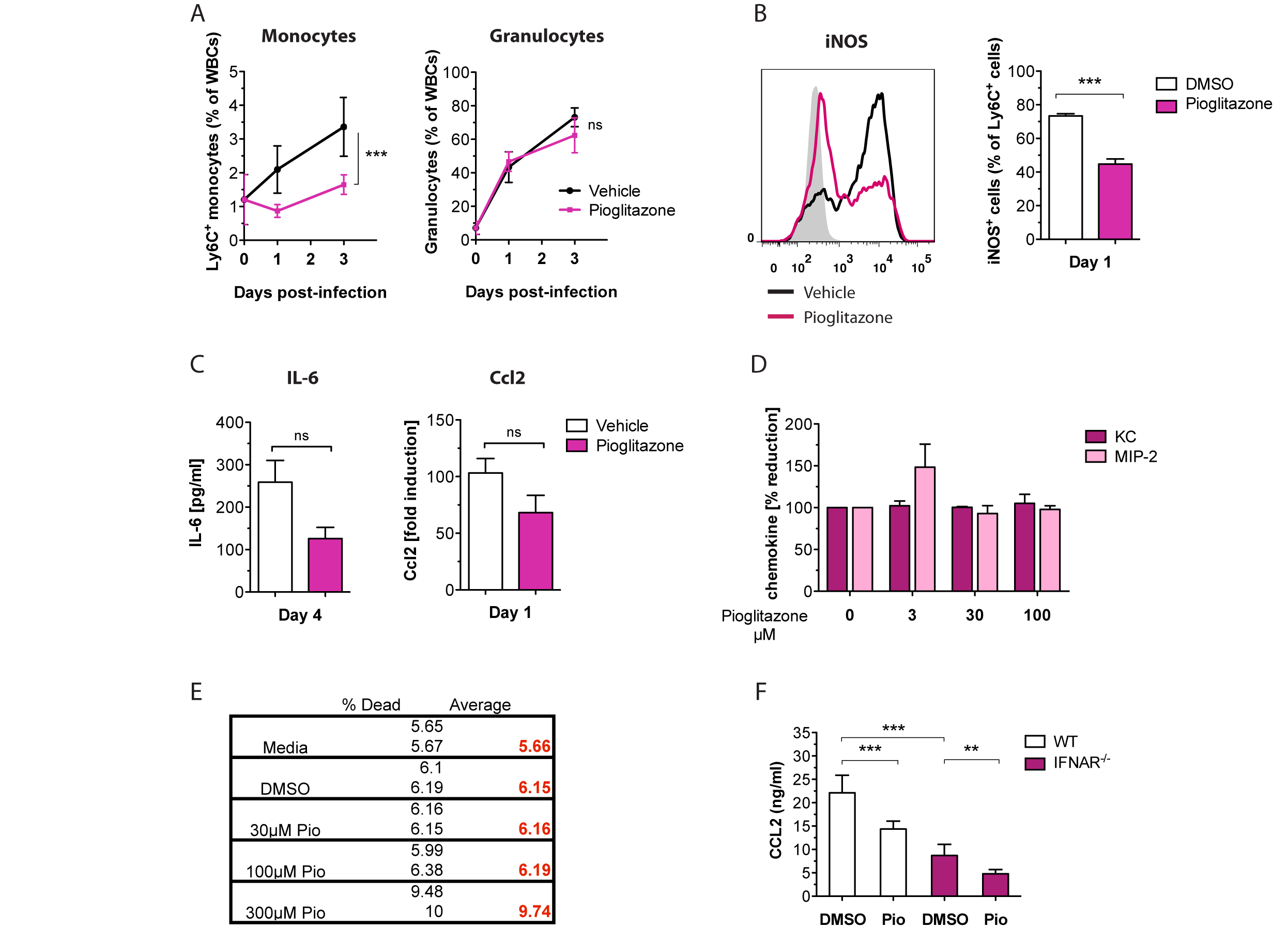

Supplement: Figure S4 — Pioglitazone attenuates inflammatory host responses. (A,C) WT mice were injected with a lethal dose of 1×10^5^ cfus Ca and treated daily with 5 mg/kg pioglitazone. At indicated time points, blood and kidneys were collected. (A) Blood samples were analyzed for the percentage of inflammatory monocytes (left) or granulocytes (right) in total WBCs. Data presented show the mean ± SD of one representative experiment out of two independent repeats (n = 3–5 mice per group). (B) BM-DCs were pre-treated with 100 µM pioglitazone overnight and stimulated the next day with heat-inactivated Ca for 24 h. Expression of iNOS was determined by intracellular staining. For analysis and histogram presentation only Ly6C+ cells have been gated. Solid lines; staining of Ly6C+ cells after Ca stimulation; shaded histograms; staining of unstimulated culture. Bar diagram shows the quantification of iNOS+ cells of total Ly6C+ cells, mean ± SD (n = 4). (C) Sera concentrations of IL-6 (left) were measured using ELISA. Kidney total RNA was analysed for gene expression of Ccl2. Data presented show the mean ± SEM (n = 5–6 mice per group). (D) BM-DCs were pre-treated overnight with indicated concentrations of pioglitazone and stimulated with heat-inactivated Ca the next day. After 24 h of Ca co-incubation, KC and MIP-2 release were measured by a multiplex bead array system. Data presented show the mean ± SEM of 3 independent experiments. (E) Cytotoxic effect of pioglitazone. BM-DCs were pre-treated with varying concentrations of pioglitazone for 24 h and cell viability was determined by live-dead staining of cells. Data presented shows one representative of two independent experimental repeats. (F) WT or Ifnar1−/− BM-DCs were pre-treated with 100 µM pioglitazone overnight and stimulated the next day with heat-inactivated Ca. After 24 h, CCL2 release was measured by ELISA. Data presented show the mean ± SD of 3 independent experiments. (TIF) [file ppat.1002811.s004.tif]
