# Supplementary material for: Type I Interferons Promote Fatal Immunopathology by Regulating Inflammatory Monocytes and Neutrophils during Candida Infections
Source: PLoS Pathog. 2012 Jul 26;8(7):e1002811. doi: 10.1371/journal.ppat.1002811 (PMC3406095; doi:10.1371/journal.ppat.1002811)
Supplement: Text S1 — Supplemental information, including 4 Figures as well as 2 Tables and additional experimental procedures. (DOC) [file ppat.1002811.s005.doc]

**Supplemental Information**

**Table S1. Markers for characterization of immune cells**

| Cell | CD11b | Ly6C | Ly6G | CD11c | iNOS | PDCA1 | CD3 | CD4 | CD8 |
| --- | --- | --- | --- | --- | --- | --- | --- | --- | --- |
| Neutrophil | + + + | + + | + + + | - | - | - |  |  |  |
| Inflammatory Monocyte | + + + | + + + | - | - / + | - / + | - / + |  |  |  |
| Inflammatory DC | + + + | + + + | - | + + + | + | + |  |  |  |
| CD4 T cell |  |  |  |  |  |  | + | + | - |
| CD8 T cell |  |  |  |  |  |  | + | - | + |

Cell surface expression levels: + + +, high level of expression; + +, intermediate expression; +, low expression; -, negative; gray-shaded cells, not applicable.

**Table S2. Oligonucleotide primers used in this study**

| Name | Sequence (5’-3’) | Reference |
| --- | --- | --- |
| *HPRT_fw* | CATAACCTGGTTCATCATCGC | qPrimer Depot^1^ |
| *HPRT_rev* | TCCTCCTCAGACCGCTTTT |
| *iNOS_fwd* | GTTCTCAGCCCAACAATACAAGA | Harvard^2^ |
| *iNOS_rev* | GTGGACGGGTCGATGTCAC |
| *KIM1_fwd* | ACATATCGTGGAATCACAACGAC | Harvard |
| *KIM1_rev* | ACAAGCAGAAGATGGGCATTG |
| *Ccl2_fwd* | ATTGGGATCATCTTGCTGGT | qPrimer Depot |
| *Ccl2_rev* | CCTGCTGTTCACAGTTGCC |
| *Ccl7_fwd* | CCACATGCTGCTATGTCAAGA | Harvard |
| *Ccl7_rev* | ACACCGACTACTGGTGATCCT |
| *ICAM1_fwd* | TGGATACCTGAGCATCACCA | qPrimer Depot |
| *ICAM1_rev* | CTGCTACCTGCACTTTGCC |
| *P-Selectin_fwd* | GAACAATCCAGGTTGCCTTG | qPrimer Depot |
| *P-Selectin_rev* | CAGTTCATGTGCGATGAAGG |
| *Il1b_fwd* | CCAACAAGTGATATTCTCCATGAG |  |
| *Il1b_rev* | TCTTTCATTACACAGGACAGGT |  |

^1^ <http://mouseprimerdepot.nci.nih.gov/>

^2^ http://pga.mgh.harvard.edu/primerbank/

**Supplemental material and methods**

**Growth conditions of Ca**

Ca was grown overnight at 30°C in rich (YPD) medium under constant shaking, and diluted to an OD_600_ of 0.2 in YPD medium the next morning, before cultivating them at 30°C under constant shaking until the logarithmic growth phase. Fungal cells were collected by centrifugation, washed in sterile PBS and fungal cell count was determined using a CASY cell counter (**Innovatis AG**). Candida cell suspensions were diluted at the required cell number in PBS for mouse infections, or in appropriate cell culture media for co-culture with innate immune cells.

**Media for cell culture**

The culture medium for BM-DCs contained high-glucose DMEM (PAA), supplemented with 10% heat-inactivated FCS (Invitrogen), 10 % GM-CSF-containing X-conditioned medium and 100 U/ml Penicillin, 100 μg/ml Streptomycin. X-conditioned media was prepared in our laboratory exactly as previously described using the GM-CSF-producing X-63 cell line .

**Cell culture of primary dendritic cells differentiated from bone marrow**

Seven to nine week-old C57BL/6 wild type or knock-out mice were used for the preparation of BM-DCs. Bone marrow was collected from mouse femurs, treated with red blood lysis buffer (8.29 g/l NH4Cl, 1 g/l KHCO3, 0.0372 g/l EDTA, pH 7.2-7.4.) and resuspended in the appropriate media to generate BM-DCs according to previously described methods . After 3 days in culture, fresh medium was added. BM-DCs were obtained after 7-8 days in culture. Cell surface markers of the BM-DCs cell preparation were assessed by flow cytometry analysis using a panel of fluorescently-labeled antibodies against cell-surface markers (BD Bioscience). For a detailed characterisation of our cell-culture model see .

**Leukocyte enrichment from mouse kidneys**

The method has been adapted from Lionakis et al. . Briefly, kidneys were weighted, finely minced and digested at 37°C in digestion solution containing Liberase TL (Roche Diagnostic) and 300U/ml of grade II DNAse I (Roche) for 20min with intermittent shaking. Digested tissue was passed through a 70-μm filter, washed, and the remaining red cells were lysed with ACK lysis buffer. Red cell-free cell suspensions were passed through a 40-μm filter, washed, and suspended in 8 ml of 40% Percoll (GE Healthcare). Leukocyte enrichment was performed by overlay of the Percoll-cell suspension on 3 ml of 70% Percoll, and centrifugation at 2,000 rpm for 30 min at RT. The leukocytes accumulating at the interphase were isolated, washed in PBS, and suspended in FACS buffer.

**Co-culture of innate immune cells with fungi**

For co-culture of immune cells with fungi, GM-CSF differentiated bone marrow cells were plated at a density of 1.0-1.25x10^5^ cells/cm^2^ one day prior to the assay, as previously described . Fungal-mammalian cell co-culture was performed at a multiplicity of infection (MOI) of 2:1 for the indicated time periods as previously described . If necessary, Ca was heat-inactivated for 10min at 70°C before interaction. In some experiments cells were pre-incubated for 16-18h with pioglitazone (dissolved in DMSO) before Ca stimulation. IFNAR1 blocking with anti-IFNAR1 Ab (Mar1-5A3, Biolegend) was performed one hour prior addition of Ca.

**Reverse transcription and real-time PCR analysis**

Total RNA was isolated from BM-DCs or kidney homogenates using a centrifugation column-based kit (Promega) according to the manufacturer’s instructions. Total RNA samples were eluted in 50µl RNase-free sterile water. RNA concentration was measured using a NanoDrop2000 (Thermo Scientific) and samples were stored at -80°C until further use. Reverse-transcription was performed using a reverse transcription kit (Promega) according to conditions recommended by the manufacturer. Typically, reactions were carried out on 0.5-1 µg total RNA with oligo-dT primers in a final volume of 20µL. Reverse transcription products were diluted 1:5 with water and stored at –20°C until further use.

For the real-time PCR amplification, 2µl of the diluted cDNAs were added to 20μl of real-time PCR mix (200nM forward primer, 200nM reverse primer, 1X KAPA SYBR FAST qPCR MasterMix (Peqlab) and submitted to the following cycling conditions: 95°C for 4min, 40 cycles (95°C for 3s, 60°C for 20s, 72°C for 8s, signal detection at 72°C) followed by a melting curve analysis.

**Protein extracts and immunoblotting**

Cells were scrapped on ice in 80µl ice-cold protein lysis buffer (10mM Tris pH7,5, 50mM NaCl, 1% Triton X-100, 1mM PMSF, 1X protease inhibitor cocktail (Roche)), collected and centrifuged at 15000xg at 4°C for 10 min. Sample supernatants were transferred to fresh tubes containing 30µl of 4X Sample Buffer, heated at 95°C for 5-7min, cooled on ice and stored at -20°C until further use. Aliquots of 15µl of proteins samples were fractionated by SDS-PAGE and transferred onto nitrocellulose membranes. Prior to immunodetection, membranes were blocked for 1-2 hours in 1X TBST with 10% non-fat dry milk. Blots were probed with anti-STAT1 antibodies recognizing phospho-Tyr701 (Cell Signaling) and anti-p38 (Cell Signaling) as loading control. Immune complexes were detected with an infrared-labeled secondary antibody (LI-Cor). Analysis was performed using the infrared imaging system Odyssey (LI-Cor), according to the manufacturer's instructions.

**Supplemental references**

1. Bourgeois C, Majer O, Frohner I, Kuchler K (2009) In vitro systems for studying the interaction of fungal pathogens with primary cells from the mammalian innate immune system. Methods Mol Biol 470: 125-139.

2. Zal T, Volkmann A, Stockinger B (1994) Mechanisms of tolerance induction in major histocompatibility complex class II-restricted T cells specific for a blood-borne self-antigen. J Exp Med 180: 2089-2099.

3. Hume DA, Gordon S (1983) Optimal conditions for proliferation of bone marrow-derived mouse macrophages in culture: the roles of CSF-1, serum, Ca2+, and adherence. J Cell Physiol 117: 189-194.

4. Inaba K, Inaba M, Romani N, Aya H, Deguchi M, et al. (1992) Generation of large numbers of dendritic cells from mouse bone marrow cultures supplemented with granulocyte/macrophage colony-stimulating factor. J Exp Med 176: 1693-1702.

5. Bourgeois C, Majer O, Frohner IE, Lesiak-Markowicz I, Hildering KS, et al. (2011) Conventional dendritic cells mount a type I IFN response against *Candida* spp. requiring novel phagosomal TLR7-mediated IFN-beta signaling. J Immunol 186: 3104-3112.

6. Lionakis MS, Lim JK, Lee CC, Murphy PM (2011) Organ-specific innate immune responses in a mouse model of invasive candidiasis. J Innate Immun 3: 180-199.
